# MacroH2A1 chromatin specification requires its docking domain and acetylation of H2B lysine 20

**DOI:** 10.1038/s41467-018-07189-8

**Published:** 2018-12-03

**Authors:** Penelope D. Ruiz, Matthew J. Gamble

**Affiliations:** 1 Department of Molecular Pharmacology, Albert Einstein College of Medicine, Bronx, NY 10461 USA; 2 Department of Cell Biology, Albert Einstein College of Medicine, Bronx, NY 10461 USA

## Abstract

The histone variant macroH2A1 localizes to two functionally distinct chromatin subtypes marked by either H3K27me3 or H2B acetylations, where it is thought to directly regulate transcription. The recent finding, that macroH2A1 regulates mitochondrial respiration by globally dampening PARP activity, requires the field to re-evaluate which functions of macroH2A1 are due to global effects on cellular metabolism and which are direct effects determined by macroH2A1 chromatin localization. Here, we demonstrate macroH2A1 incorporation into H2B-acetylated chromatin requires a feature in its histone-fold domain, distinguishing this process from incorporation into H3K27me3-containing chromatin in which multiple features of macroH2A1 are sufficient for targeting. In addition, we identify H2BK20 acetylation as a critical modification required to target macroH2A1 to H2B-acetylated chromatin. Our findings have allowed us to definitively establish that macroH2A1’s regulation of an important transcriptional program, the senescence-associated secretory phenotype (SASP), requires its accurate genomic localization.

## Abstract (web summary)

The histone variant macroH2A1 localizes to two functionally distinct chromatin subtypes marked by either H3K27me3 or H2B acetylations. Here the authors identify the features of macroH2A1 required for its recruitment to H2B-acetylated chromatin and identify H2BK20 acetylation as a critical requirement for this recruitment.

## Introduction

Chromatin, made of repeating units of nucleosomes, can be locally, structurally and functionally differentiated by a variety of mechanisms including covalent post-translational modifications (PTMs) of histones, ATP-dependent remodeling and the incorporation of specialized histone variants, to regulate DNA-dependent reactions^1^. Unlike their canonical counterparts, histone variants are incorporated in a replication-independent manner to perform a particular function predicated on its targeting to the appropriate genomic locations. There are two fundamental requirements for this targeting. (1) The histone variant must harbor a specific feature differentiating it from other histones. (2) A feature at the site of chromatin incorporation must serve as a beacon to recruit the histone variant to the appropriate genomic locations.

MacroH2A-type histone variants, which include macroH2A1.1, macroH2A1.2, and macroH2A2, are composed of a histone-fold domain (HFD), a basic linker region and a 25 kDa carboxyl-terminal region called a macrodomain^2^. At three-times the size of canonical H2A, macroH2A-containing nucleosomes organize the same amount of DNA^2^. The linker and macrodomain emerge from the nucleosome core where they participate in the recruitment of a host of co-factors^3–9^. Generally, macroH2As create large, specialized chromatin environments, hundreds of kilobases long^4,10^. While the overall structure of the three macroH2A isoforms is conserved, an alternative splicing event renders macroH2A1.1 capable of binding the PTM poly(ADP-ribose)(PAR) produced by PAR polymerases (PARPs), which plays key roles in the ability of macroH2A1.1 to regulate gene transcription^4,11^ and DNA damage responses^12^. Two critical glycines (Gly224 and Gly314) are required for PAR binding^11^. The macrodomains of macroH2A1.2 and macroH2A2 lack these critical glycines and contain a three amino acid insertion which collectively disrupts the stabilizing interactions observed between PAR and macroH2A1.1^11,13^. The relative level of macroH2A1.1 splicing is significantly perturbed across many types of cancer, resulting in reduced macroH2A1.1 expression^14–16^. Re-expression of macroH2A1.1 suppressed cancer cell proliferation^14,17,18^, suppressed anchorage-independent growth and cell invasiveness in breast cancer^19^, and suppressed metastasis of melanoma in mice^18^. MacroH2A1.1 is thus not only a structural component of chromatin, but also a reader of a functionally diverse PTM with roles in transcriptional regulation and DNA damage responses.

First identified as enriched on the inactive X chromosome (Xi), macroH2As were considered markers of transcriptionally repressed chromatin^13,20^. However, later work demonstrated that macroH2A’s roles in the cell are not so simplistic. The majority of macroH2A is found on autosomes as part of facultative heterochromatin marked by trimethylation of histone H3 on lysine 27 (H3K27me3) or as part of transcriptionally active euchromatin marked by nine histone acetylations (e.g. H2B at K15 and K20; H3 at K4, K14 and K18; H4 at K91; and H2A at K5) where it can either positively or negatively regulate transcription^4,5,10,21,22^. The physiological functions of macroH2A are an active area of research; it serves as a barrier to stem cell reprogramming^23–25^, as a regulator of gene expression^22,26–28^ during normal growth and cellular senescence^29,30^. MacroH2A1.1 plays a specialized role in transcriptional regulation; through interaction with PAR, macroH2A1.1 recruits PARP1 and CBP, leading to the acetylation of H2B K12 (H2BK12ac) and K120 (H2BK120ac)^4,29^. This mechanism plays a critical role in oncogene-induced senescence (OIS), an important tumor suppressive mechanism. In OIS, macroH2A1 serves as a component of the senescence-associated heterochromatic foci involved in transcriptional repression^30^. However, macroH2A1.1 is specifically required for the transcriptional activation of a myriad of cytokines, chemokines and metalloproteases that make up the senescence-associated secretory phenotype (SASP), which enables the clearance of senescent cells^31^.

Recently, a global role for macroH2A1.1 in the regulation of mitochondrial function and energy metabolism has been identified. Through its interaction with PAR and concomitant inhibition of PARP1 activity, macroH2A1.1 allows for greater production of NAD^+^ in mitochondria^32^ suggesting that macroH2A1 plays a global role in cellular energetics, regardless of where macroH2A1 localizes on the genome. This result requires the field to carefully re-evaluate which functions of macroH2A1 are mediated by global effects of macroH2A1 on cellular energetics and which require it to function locally through deposition at specific chromatin loci.

Here, we demonstrate that the requirements for deposition of macroH2A1 into H2Bac and H3K27me3-marked chromatin are distinct. We find that the carboxyl-terminal region of the HFD of macroH2A1 is required for association with H2B-acetylated chromatin, whereas multiple features of macroH2A1 are sufficient for association with chromatin marked by H3K27me3. Furthermore, we determined that regulation of macroH2A1-dependent transcriptional programs, such as the SASP, requires its ability to accurately localize to its H2B-acetylated chromatin niche. Additionally, we identified H2BK20ac as a key PTM which serves as a beacon to target macroH2A1 into the H2B-acetylated chromatin environment. The loss of macroH2A1 from H2B-acetylated chromatin, caused either by mutating macroH2A1 itself or by ablating the H2BK20ac chromatin cue for macroH2A1 deposition, led to a defect in the upregulation of SASP transcription. Overall, our work highlights key elements regulating the deposition of macroH2A1 into H2B-acetylated chromatin and demonstrates that macroH2A1 plays critical local roles in regulating gene expression.

## Results

### Ectopic wild-type macroH2A1 incorporates into nucleosomes

**Fig. 1:**
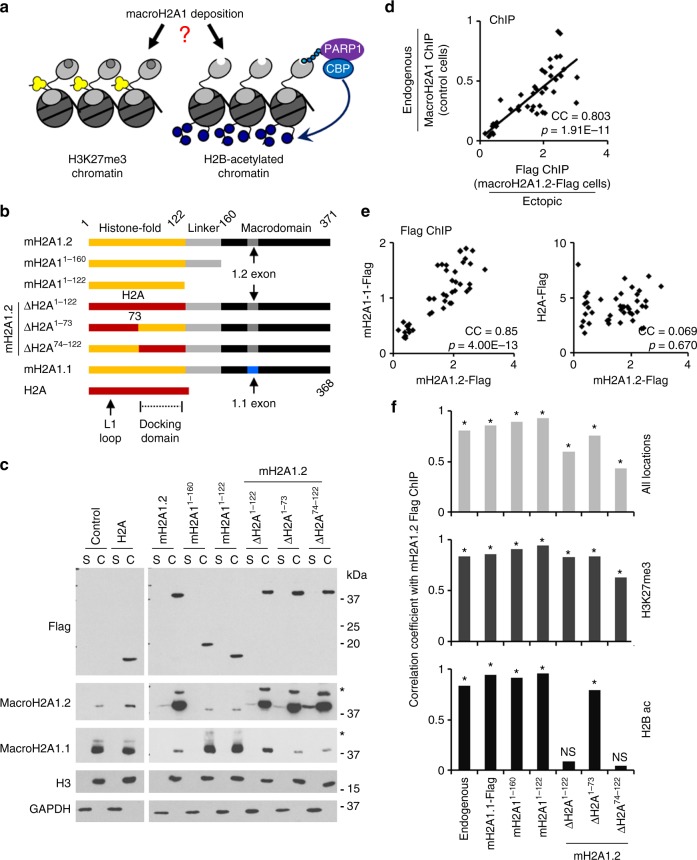
MacroH2A1 requires the carboxyl-terminal half of its HFD for accurate deposition into H2B-acetylated chromatin. **a** Schematic depicting the different chromatin environments into which macroH2A1 is deposited marked by either H3K27me3 or acetylations at H2B K12, K15, K20, and K120. In H2B-acetylated chromatin, macroH2A1.1 cooperates with PARP1 through binding PAR to recruit CBP and regulate transcription. **b** Diagram of mutant macroH2A1.2-Flag (mH2A1) constructs expressed in IMR90-hTERT cells. The macroH2A1 alternative exons a nd docking domain are indicated. **c** Immunoblots of soluble (S) and chromatin (C) fractions from IMR90-hTERT cells described in **b** for indicated proteins. The minor band (*) above the major band in the macroH2A1.1 and macroH2A1.2 immunoblots is the mono-ubiquitylated species of macroH2A1. **d** Scatter plot of Flag ChIP from wild-type macroH2A1.2-Flag cells plotted against macroH2A1 ChIP from control cells expressing GFP (*n* = 3 independent cell passages). Pearson’s correlation coefficient (CC) and associated p-value indicated are from Pearson’s product-moment correlation. **e** Scatter plot of Flag ChIP from wild-type macroH2A1.2-Flag cells versus Flag ChIP from H2A-Flag cells. (*n* = 3 independent cell passages). **f** Pearson’s correlation coefficient calculated for Flag ChIP from wild-type macroH2A1.2-Flag cells versus vs. Flag ChIP from cells expressing macroH2A1.2-Flag truncations and domain swap mutants described in **b**. Top graph contains primers across both types or chromatin. Middle graph contains primers for regions marked with H3K27me3. Bottom graph contains primers for regions marked with H2Bac. (*n* = 3 independent cell passages, *p < 0.003 from Pearson’s product-moment correlation, NS not significant)

While several groups, including ours, have determined where macroH2A1 localizes across the genome in several cell types^4,25,33–35^, we know little about the rules governing specification of macroH2A1-containing chromatin. Previous studies have demonstrated that multiple features of macroH2A1 were sufficient to target the histone variant to the predominantly H3K27me3-containing Xi, including regions in macroH2A1’s HFD and macrodomain^36,37^. Subsequently, we determined that macroH2A1 localized to H3K27me3-containing chromatin across autosomes as well^10^. Additionally, we determined that a similar proportion of macroH2A1 localizes to transcriptionally active chromatin (Fig. 1a)^4^.

These additional findings led us to re-evaluate the rules that dictate macroH2A1 genomic localization. Using our expanded knowledge of macroH2A1 localization and a series of macroH2A1 domain deletion and substitution mutants, we sought to identify the features of macroH2A1 required to specify its accurate deposition into its two chromatin subtypes, H3K27me3-marked chromatin or H2Bac-marked chromatin. To this end, we derived a panel of cell lines from immortalized IMR90 human diploid lung fibroblasts that expressed a mutant macroH2A1 protein harboring a carboxyl-terminal Flag-tag (macroH2A1-Flag). Ectopic expression of macroH2A1.1 has been shown to induce cellular senescence^29^, so ectopically expressed full-length macroH2A1 constructs possessed the macroH2A1.2 macrodomain to establish stable, replicative cell lines. Two lines harbored truncation mutants lacking the macrodomain alone (macroH2A1^1–160^-Flag) or lacking both the linker and macrodomain (macroH2A1^1–122^-Flag) (Fig. 1b). In an additional macroH2A1 mutant the HFD of macroH2A1.2 was replaced with the homologous region of canonical H2A (macroH2A1.2^ΔH2A1–122^-Flag). To ensure that each ectopic macroH2A1 mutant and wild-type protein could incorporate into chromatin, we fractionated the cells into soluble and chromatin fractions and liberated the mononucleosomes from the chromatin fraction with Micrococcal nuclease (MNase) (Fig. 1c). A Flag immunoblot indicated that the overwhelming majority of the ectopically expressed macroH2A1.2-Flag proteins were chromatin incorporated, similar to endogenous macroH2A1 and H3.

We previously described a compensatory post-translational feedback mechanism which decreases the level of endogenous macroH2A1 when macroH2A1 is ectopically expressed^4^. The feedback and reduction of macroH2A1.1 can be clearly observed in all the ectopic macroH2A1.2-Flag mutant lines that harbor an intact macrodomain (Fig. 1c); however, the macroH2A1-Flag lines lacking the macrodomain (e.g., macroH2A1^1–160^ and macroH2A1^1–122^) failed to reduce endogenous macroH2A1.1 indicating that the macrodomain plays a critical role in this compensatory feedback loop.

### Multiple features of macroH2A1 target it to H3K27me3

Having ensured that ectopic macroH2A1-Flag proteins incorporate into chromatin and do not significantly alter nuclear size (Supplementary Fig. 1a–b), we turned our attention to determining where they were being deposited using Flag chromatin immunoprecipitation assayed by quantitative PCR (ChIP-qPCR). Using our previous ChIP-seq data^4^ and publicly available data^38^, we designed ChIP primers targeting regions with and without macroH2A1 enrichment in both H2Bac and H3K27me3-containing chromatin (Supplementary Table 1). Data from endogenous macroH2A1 ChIP in parental cells highly correlated to data from a Flag ChIP from cells ectopically expressing wild-type macroH2A1.2-Flag, demonstrating that ectopically expressed wild-type macroH2A1.2 was genomically targeted in a manner similar to the endogenous protein (Fig. 1d, Supplementary Fig. 2). The correlation remained high when looking specifically at association with either H2Bac chromatin or H3K27me3-marked chromatin indicating that ectopic wild-type macroH2A1.2 correctly localizes to both chromatin environments (Fig. 1f). Consistent with a distinct pattern of deposition, there was no significant correlation between the Flag ChIPs from cells expressing macroH2A1.2-Flag and canonical H2A-Flag (Fig. 1e, Supplementary Fig. 2).

Armed with the Flag ChIP-qPCR approach, we sought to determine which macroH2A1 domains are required to correctly specify macroH2A1-containing chromatin. When we compared the Flag ChIP data from wild-type macroH2A1.2-Flag expressing cells with data from cells expressing macroH2A1^1–160^-Flag, lacking the macrodomain, or macroH2A1^1–122^-Flag, lacking the linker region and macrodomain, we found that either mutant demonstrated a high correlation with wild-type macroH2A1.2-Flag, indicating that neither of those domains are required for the specification of macroH2A1-containing chromatin and that the HFD of macroH2A1 is sufficient to drive this process (Fig. 1f).

To test the role of the HFD in the specification of macroH2A1-containing H3K27me3 chromatin, we performed Flag ChIP on cells expressing macroH2A1.2^ΔH2A1–122^-Flag. This mutant correlated well with wild-type macroH2A1.2-Flag ChIP, indicating that the HFD of macroH2A1.2 is also not required for targeting its deposition into H3K27me3-marked chromatin (Fig. 1f, Supplementary Fig. 3). The fact that all three domain mutants could localize to H3K27me3-marked chromatin in a manner similar to wild-type indicates that multiple features of macroH2A1 can target its deposition to H3K27me3-marked chromatin. Overall, this is consistent with previous studies on the localization of macroH2A1 to the H3K27me3-marked Xi^36,37^.

A previous report showed that replacing the L1 loop and α1 helix of canonical H2A with the homologous regions of macroH2A1 in the absence of the macroH2A1 linker and macrodomain was sufficient for enrichment on the inactive X^36^. We expressed a chimera containing the N-terminal half of macroH2A1, possessing the macroH2A1 L1 loop and α1 helix, fused to the C-terminal half of canonical H2A (macroH2A1-HFD^ΔH2A73–122^-Flag). Our results confirmed the previous finding showing that this chimera was accurately deposited into autosomal H3K27me3-containing chromatin (Supplementary Fig. 3). Together our results extend earlier findings by demonstrating that multiple regions of macroH2A1 are sufficient to target this histone variant to H3K27me3-marked autosomal heterochromatin in addition to the previously described H3K27me3-marked Xi.

### Critical role of the HFD in specifying macroH2A1 localization

Previously, our lab demonstrated that most macroH2A1-regulated genes in IMR90 cells are found in chromatin co-occupied by macroH2A1 and H2Bac, making this type of chromatin particularly important for macroH2A1’s functions^4,29^. MacroH2A1 mutants lacking the macrodomain or the macrodomain and linker domain highly correlate with wild-type macroH2A1.2 association in H2Bac chromatin (Fig. 1f). Accordingly, neither domain is required for macroH2A1 association with H2Bac chromatin. However, the macroH2A1.2^ΔH2A1–122^-Flag mutant, where the HFD was replaced with canonical H2A, does not show a significant correlation with wild-type macroH2A1.2-Flag in H2Bac chromatin (Fig. 1f). This indicates a specific requirement for the HFD in targeting macroH2A1 to H2Bac chromatin, differentiating it from the rules governing macroH2A1 association with H3K27me3-marked chromatin.

The HFD of macroH2A1 is more highly conserved across vertebrates than canonical H2A^39^. Amino acid sequence alignment between H2A-type histones indicate regions of difference between macroH2A and the other H2A-type histones that may confer specificity to macroH2A chromatin deposition (Supplementary Fig. 4). Two key regions of divergence are the L1-loop and the docking domain. The L1-loop creates the only interface between the two H2A-H2B dimers within a single nucleosome. The structurally distinct L1-loop of macroH2A1 regulates the preference of macroH2A1 to form heterotypic nucleosomes with canonical H2A^2,40^. The H2A docking domain, composed of two short helices and containing a conserved acidic patch, interacts with the H3/H4 tetramer and locks the H3 α-N helix in position^41^. MacroH2A1 and canonical H2A docking domains harbor several amino acid differences, but demonstrate high structural similarity^2^. The docking domain of H2A.Z, specifically the acidic patch, was shown to be required for H2A.Z deposition and its function^42,43^.

To determine which feature was dominant in specifying macroH2A1-containing-H2Bac chromatin, we used a chimera-based approach similar to one used to identify regions of H2A.Z critical for its correct localization^43,44^. Each half of the HFD of macroH2A1.2 was replaced with the homologous region of canonical H2A (Fig. 1b). Replacement of the amino-terminal half of the HFD of macroH2A1 with canonical H2A (macroH2A1.2^ΔH2A1–73^-Flag) did not disrupt specification of macroH2A1-containing chromatin. However, replacement of the carboxyl-terminal half of the HFD of macroH2A1 with the homologous region of canonical H2A (macroH2A1.2^ΔH2A74–122^-Flag) failed to significantly correlate with wild-type macroH2A1.2, specifically in the H2Bac-marked chromatin (Fig. 1f) as did a similar chimera lacking the macrodomain (Supplementary Fig. 2). Overall, this indicates that the carboxyl-terminal half of macroH2A1’s HFD is required for accurate deposition of this histone variant into its H2Bac chromatin environment.

### MacroH2A1 localization regulates transcriptional program

**Fig. 2:**
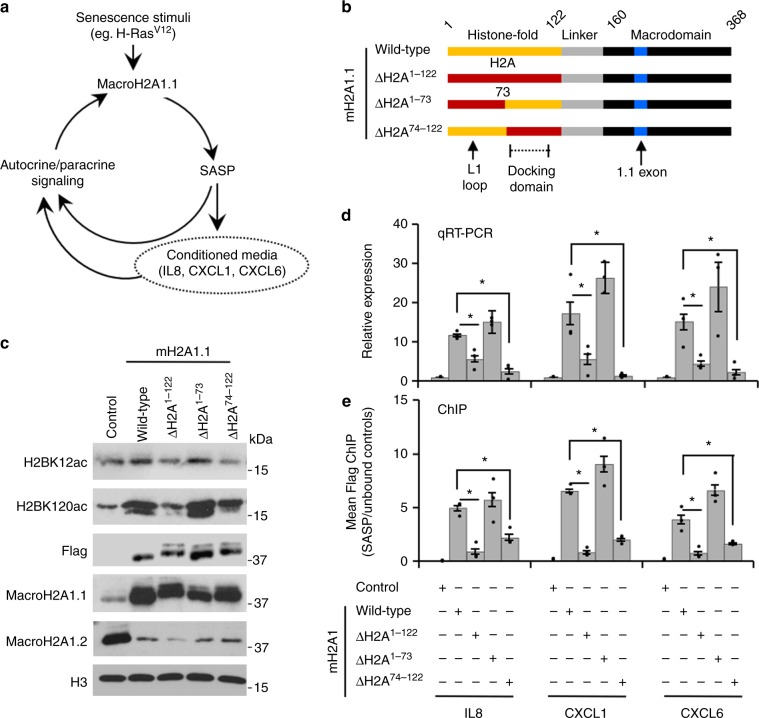
MacroH2A1 localization to H2Bac chromatin is required for regulation of H2BK12ac, H2BK120ac, and SASP gene transcription. **a** Diagram illustrates macroH2A1.1-dependent positive feedback loop regulating the induction of SASP gene expression. Senescent stimuli, such as H-Ras^V12^ upregulates the expression of macroH2A1.1 which promotes the transcription of SASP genes. Secreted SASP factors can act in an autocrine or paracrine fashion. Conditioned media from senescent cells contains secreted cytokines and chemokines (SASP factors) which can promote senescence through paracrine signaling. **b** Schematic of domain swap macroH2A1.1-Flag (mH2A) mutant lentiviral constructs expressed in IMR90-hTERT cells. MacroH2A1.1 exon and docking domain indicated. **c** Immunoblots of acid-extracted histones for indicated histones and histone PTMs from cells that ectopically express macroH2A1.1 constructs described in **b**. **d** RT-qPCR for SASP factors of cells described in **b**. Error bars, s.e.m. (*n* = 3 independent lentiviral infections, *p < 0.05 from a two-tailed Student’s *t*-test). **e** Flag ChIP-qPCR in macroH2A1.2-Flag cells for SASP genes shown in **d**. Bars represent enrichment of macroH2A1-Flag at SASP loci over unbound control regions. Error bars, s.e.m. (*n* = 3 independent cell passages, *p < 0.05 from a two-tailed Student’s *t*-test)

Cellular senescence, triggered by telomere dysfunction, oncogenic stress, or DNA damage, results in stable withdrawal from the cell cycle through the engagement of tumor suppressor pathways^31^. An additional feature of senescence is the SASP, a transcriptional program inducing the expression of a host of secreted inflammatory factors that contribute to recruitment of innate immune effectors and reinforce the senescent state through paracrine signaling (Fig. 2a)^31^. SASP genes reside in macroH2A1-containing, H2Bac-marked chromatin and require macroH2A1.1-mediated recruitment of PARP1 and the acetyltransferase CBP for their activation^29^. Our results strongly suggested that macroH2A1.1 plays a local and direct role in the transcriptional activation of SASP genes. However, recently it was shown that macroH2A1.1, through its ability to inhibit PARP1, also modulates the level of NAD^+^ available for mitochondrial respiration and global energy metabolism^32^. These findings lead to an important question: Is the regulation of transcription by macroH2A1.1 a direct effect of a local association and function of macroH2A1.1 at the genes it is transcriptionally regulating or is it a downstream consequence of macroH2A1.1’s ability to regulate cellular metabolism?

To answer this question, we generated three mutants described above in the context of macroH2A1.1-Flag (e.g., macroH2A1.1^ΔH2A1–122^, macroH2A1.1^ΔH2A1–73^, macroH2A1.1^ΔH2A74–122^) with the knowledge that replacement of the entire HFD or the carboxyl-terminal half of the HFD leads to aberrant macroH2A1 localization (Fig. 2b). Importantly, there is a strong correlation between the deposition of ectopically expressed macroH2A1.1_Flag and ectopically expressed macroH2A1.2_Flag (Fig. 1e, f, Supplementary Fig. 2) indicating the two macroH2A1 isoforms occupy similar regions in the genome. Immunoblotting for Flag and macroH2A1.1 indicated that the ectopic macroH2A1.1 proteins were similarly expressed (Fig. 2c) and chromatin incorporated (Supplementary Fig. 5). As we have shown previously^29^, ectopic expression of wild-type macroH2A1.1-Flag induced SASP gene expression as assayed by monitoring three SASP genes (e.g., IL8, CXCL1, and CXCL6) by RT-qPCR. The results demonstrated that localization deficient macroH2A1.1-Flag mutants, macroH2A1.1^ΔH2A1–122^ and macroH2A1.1^ΔH2A74–122^, were defective in their ability to induce SASP gene expression (Fig. 2d). As a control, we confirmed the ability of macroH2A1.1^ΔH2A1–73^ to correctly localize to the SASP genes by performing Flag ChIP (Fig. 2e). Overall, these results highlight the importance of the local function of macroH2A1, and demonstrate that macroH2A1.1 localization to its target genes is critical to regulate their transcription.

### MacroH2A1.1 chromatin targeting regulates H2BK12ac and K120ac

We previously demonstrated that macroH2A1.1, in conjunction with PARP1, recruits the acetyltransferase CBP to promote H2BK12ac and H2BK120ac^4^. When either wild-type macroH2A1.1 or macroH2A1.1^ΔH2A1–73^, which can correctly localize to chromatin, is ectopically expressed, this regulation manifests as an increase in total H2BK12ac and H2BK120ac levels (Fig. 2c). The effect of macroH2A1.1 on these PTMs is abrogated in the macroH2A1.1 localization defective mutants, macroH2A1.1^ΔH2A1–122^ and macroH2A1.1^ΔH2A74–122^ (Fig. 2c). Together these data indicate that macroH2A1.1 must be localized to its appropriate chromatin niche to promote H2BK12ac and H2BK120ac. We hypothesized there must be additional, yet unidentified, factors at the endogenous chromatin niche that influence the ability of macroH2A1.1 to regulate the CBP-mediated acetylation of H2B at K12 and K120, and subsequently macroH2A1-dependent gene transcription.

### Defining the macroH2A1-containing H2Bac chromatin niche

**Fig. 3:**
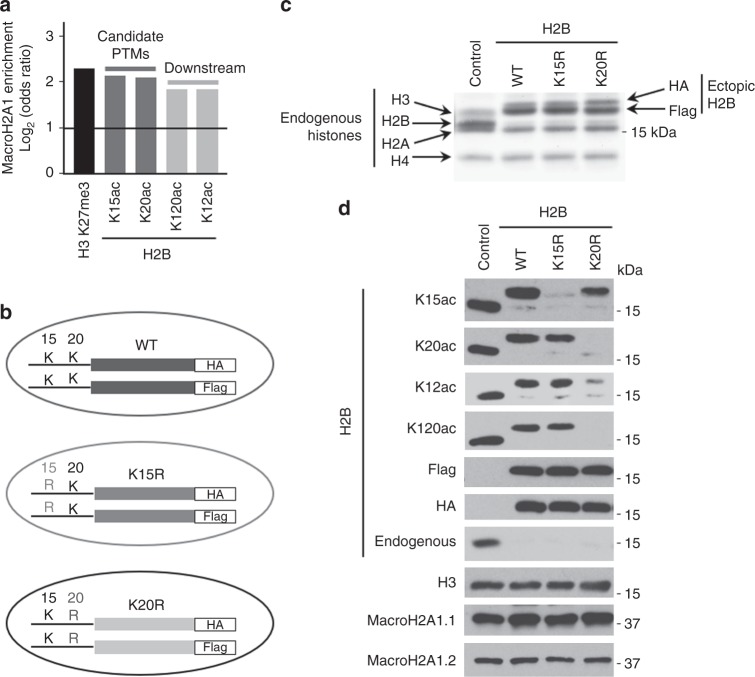
H2BK20ac is required for acetylation of H2B on K12 and K120. **a** ChIP-seq data for macroH2A1 and 5 histone PTMs^4^ enriched for macroH2A1 in IMR90 cells. **b** Schematic for co-expression of two ectopic H2B constructs in IMR90-hTERT cells to ablate H2BK15ac and H2BK20ac. (WT, wild-type). **c** Coomassie-stained gel of acid-extracted histones from cells described in **b**. Bands corresponding to histones are indicated. **d** Immunoblots of acid-extracted histones from cell lines described in **b** for indicated H2B PTMs and proteins

We next sought to uncover the additional features of the macroH2A1-containing H2Bac chromatin niche required for macroH2A1.1 to promote H2BK12ac and H2BK120ac and thereby regulate target gene transcription. Of the nine acetylations enriched in macroH2A1-containing chromatin^4^, only two of those acetylations, H2BK15ac and H2BK20ac, exhibit a stronger genome-wide enrichment for macroH2A1 than the acetylations regulated by macroH2A1.1 (e.g., H2BK12ac and H2BK120ac) (Fig. 3a), leading us to hypothesize that H2BK15ac and/or H2BK20ac play a critical role in macroH2A1’s local chromatin environment, impacting its function.

To evaluate the role of H2BK15ac and H2BK20ac in the macroH2A1 chromatin niche, we ablated these marks in IMR90 cells. Depleting H2BK15ac and H2BK20ac through enzyme inhibition, shRNA or CRISPR/Cas9 strategies is impractical as multiple promiscuous enzymes are capable of acetylating these sites^45–47^. Therefore, we decided to replace endogenous H2B with ectopically expressed H2B harboring lysine-to-arginine mutations at position 15 or 20 to render these sites incapable of acetylation. To replace endogenous H2B, a highly expressed protein, we generated stable cells simultaneously expressing two H2B lentiviral constructs with identical mutations (e.g., K15R or K20R) and either a carboxyl-terminal Flag or HA tag (Fig. 3b). A Coomassie-stained gel of total histones extracted from these cells shows that the wild-type and mutant H2B constructs replaced the overwhelming majority of endogenous H2B (Fig. 3c). We further validated the reduction of endogenous H2B using an antibody which recognizes endogenous H2B but does not react with H2B harboring a carboxy-terminal tag (Fig. 3d). As expected, cells expressing the H2B^K15R^ showed a loss of H2BK15ac, while cells expressing H2B^K20R^ showed a loss of H2BK20ac (Fig. 3d). Importantly, there was no observable crosstalk between these two marks, as loss of H2BK15ac did not affect the level of H2BK20ac and vice versa. Furthermore, cells harboring either the H2B^K15R^ or H2B^K20R^ mutants did not show altered levels of macroH2A1.1 or macroH2A1.2 (Fig. 3d).

With controls in place, we examined the effect of the H2B mutants on H2BK12ac and H2BK120ac. While there was no effect on either mark in cells expressing ectopic H2B^K15R^, there was a striking reduction in both H2BK12ac and H2BK120ac in cells expressing the H2B^K20R^ mutant histone (Fig. 3d). Overall, this indicates that H2BK20ac is required for the acetylation of H2B at K12 and K120 and implicates this histone modification in the broader function of macroH2A1.

### H2BK20ac promotes the accurate localization of macroH2A1

**Fig. 4:**
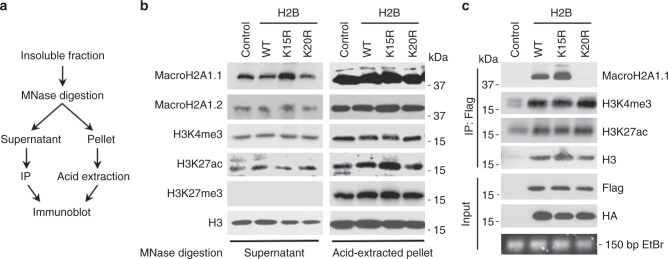
MacroH2A1.1 associates with H2B K20 acetylation in mononucleosomes. **a** Schematic for micrococcal nuclease (MNase) digestion and fractionation of euchromatic mononucleosomes for immunoprecipitation (IP) using Flag antibody. **b** Immunoblots from fractionated MNase digested cells expressing H2B mutants described in (Fig. 3b) from the supernatant and acid-extracted pellet for indicated histones and histone PTMs. **c** Immunoblots for indicated histones and histone PTMs of mononucleosome Flag IP and input material from cells described in (Fig. 3b). Bottom box is ethidium bromide stained DNA band from MNase generated mononucleosomes. Three independent MNase digestions from distinct cell passages and Flag IPs demonstrated equivalent results

We hypothesized that H2BK20ac functions as a beacon to specify macroH2A1 localization to its H2B-acetylated chromatin environment. To test this hypothesis, we liberated mononucleosomes from chromatin of ectopic wild-type H2B, H2B^K15R^ and H2B^K20R^ expressing cells using MNase (Fig. 4a). As we were most interested in the association of macroH2A1 with euchromatic, H2B-acetylated chromatin, we employed a mononucleosome preparation protocol which primarily yields euchromatic mononucleosomes^48^. The euchromatin nature of the liberated mononucleosomes was verified by the presence of euchromatic markers, such as H3K4me3 and H3K27ac, and the absence of the heterochromatic H3K27me3 (Fig. 4b). We used Flag antibody to immunoprecipitate only mononucleosomes containing the ectopic wild-type and mutant H2Bs (Fig. 4c). Cells harboring the H2B^K15R^ mutant consistently demonstrated increased levels of macroH2A1.1 liberation upon MNase digestion (Fig. 4b) and nucleosomes harboring ectopic wild-type H2B or H2B^K15R^ demonstrated a robust enrichment of macroH2A1.1. However, mononucleosomes containing H2B^K20R^ failed to incorporate macroH2A1.1 (Fig. 4c), suggesting a critical role for H2BK20ac in mediating macroH2A1 incorporation.

**Fig. 5:**
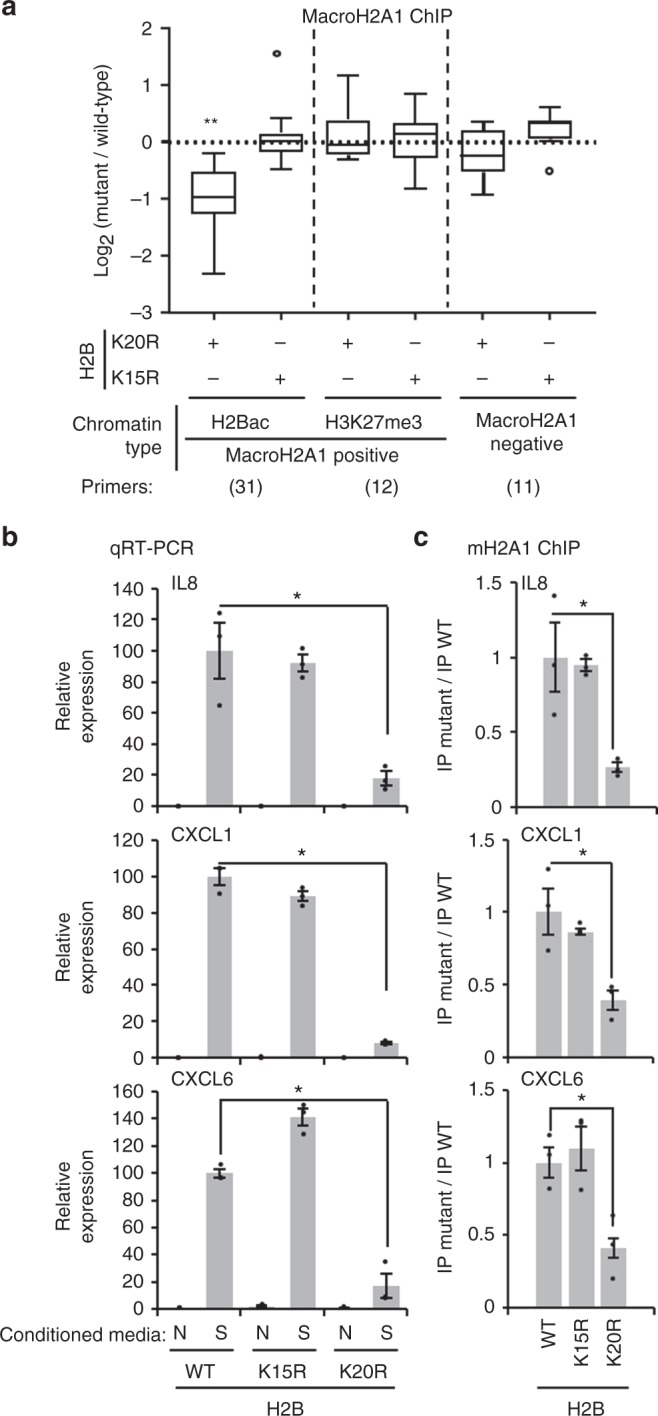
H2BK20ac promotes macroH2A1 deposition into H2B-acetylated chromatin. **a** Endogenous macroH2A1 ChIP-qPCR from cells expressing H2B constructs described in Fig. 3b using primers targeting locations within the indicated types of chromatin (regions containing either H3K27me3 or H2B-acetylated chromatin that are associated with macroH2A1 or lack macroH2A1). Data is displayed as a ratio of mutant H2B to wild-type H2B. Center line is the mean and bounds are 95% confidence interval of the mean. (*p = 1.6 × 10^−11^). **b** RT-qPCR for the indicated SASP genes from cells described in Fig. 3b that were subjected to paracrine senescence with conditioned media from H-Ras^V12^-mediated oncogene induced senescent cells (S) or with cultured media from normal cells (N) as a control. (*p < 0.02). **c** Endogenous macroH2A1 ChIP-qPCR for SASP genes shown in **b**. Bars represent the ratio of macroH2A1 association in mutant to wild-type H2B. (*p* < 0.03). Error bars, s.e.m., *n* = 3 independent cell passages, *p*-values calculated from a two-tailed Student’s *t*-test

To confirm and extend these results, we measured the relative amount of macroH2A1 localizing to both its H2Bac and H3K27me3 subtypes using endogenous macroH2A1 ChIP in the ectopic H2B expressing cell lines. Consistent with the results described above, macroH2A1 association with H2B-acetylated euchromatin sites was significantly reduced in cells expressing H2B^K20R^ compared to wild-type H2B (Fig. 5a). However, macroH2A1 association with H2B-acetylated chromatin sites were unaltered in cells expressing ectopic H2B^K15R^. No significant difference was seen in the levels of endogenous macroH2A1 associated with H3K27me3-marked, macroH2A1-containing chromatin or in regions typically devoid of macroH2A1 (Fig. 5a). Overall, these results demonstrate that H2BK20ac is critical for targeting macroH2A1 to its H2B-acetylated, euchromatic chromatin subtype.

### H2BK20ac is required for macroH2A1-regulated transcription

Earlier we demonstrated, using macroH2A1 mutants, that correct localization of macroH2A1 is critical for the transcriptional regulation of SASP genes (Fig. 2d). Armed with an understanding of the role of H2BK20ac in macroH2A1 localization, we sought to confirm this result with an independent approach. SASP gene expression can be robustly triggered through paracrine signaling whereby cytokines produced by senescent cells initiate SASP activation in neighboring cells^31,49,50^. We applied conditioned media from cells induced to senescence by oncogenic Ras^V12^ to our ectopic H2B cell lines described above without altering nuclear size (Supplementary Fig. 1c, d). Cells expressing ectopic wild-type H2B or H2B^K15R^ demonstrated a robust activation of SASP gene transcription in response to senescent cell-generated conditioned media (Fig. 5b). However, cells expressing H2B^K20R^ showed a dramatic and highly significant reduction in their ability to activate SASP gene transcription in response to senescent cell-generated conditioned media. We confirmed by ChIP that macroH2A1 association with SASP genes assayed was significantly reduced specifically in H2B^K20R^ expressing cells (Fig. 5c). Together, these results highlight the requirement for macroH2A1 to function in a specific chromatin context and demonstrate that the regulation of macroH2A1 transcriptional programs such as the SASP requires the local function of macroH2A1.

### H2BK20ac is a beacon directing macroH2A1 deposition

**Fig. 6:**
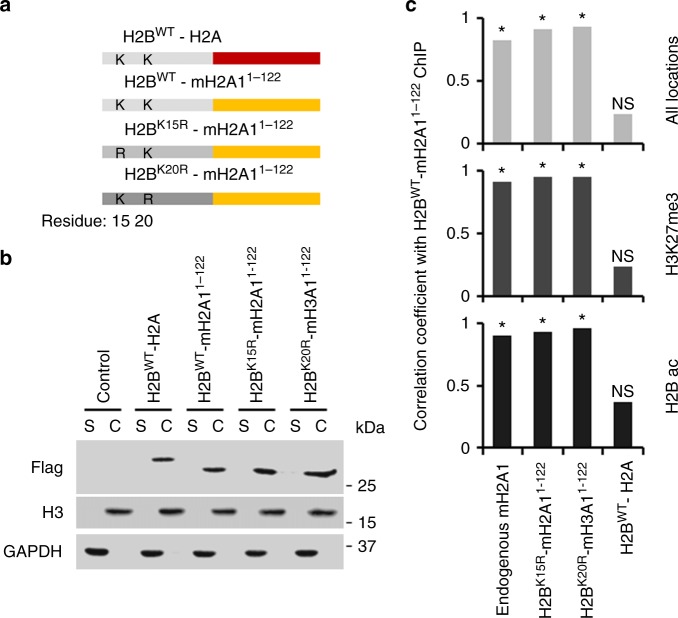
H2BK20ac is a beacon targeting macroH2A1 deposition into H2B-acetylated chromatin. **a** Schematic for single chain constructs with H2B fused directly to H2A-type histone representing obligate H2B-H2A dimers. **b** Immunoblots of soluble (S) and chromatin fractions (C) from IMR90 cells described in **a** for indicated proteins. Cells were fractionated with detergent. Insoluble fraction was digested to mononucleosomes with MNase to assess competence for chromatin incorporation. **c** Pearson’s correlation coefficient calculated for Flag ChIP in H2B^WT^-mH2A1^1–122^-Flag expressing cells versus endogenous macroH2A1 ChIP in H2B^WT^-H2A-Flag expressing cells and versus Flag ChIP in single chain obligate histone dimer expressing cells described in **a**. (*n* = 3 independent cell passages, *p < 1 × 10^−10^ from Pearson’s product-moment correlation, NS = not significant)

H2A and H2B-type histones are folded together as obligate dimers^51^. Consequently, H2A/H2B dimers are the physiological substrate for histone exchange machinery rather than monomers. Histones can be modified prior to deposition and certain modifications have been shown to be required for chromatin assembly^52,53^. We expressed H2A and H2B as a single-chain (one peptide) (Fig. 6a) using a similar strategy to one used in yeast^54^, to determine if H2BK20ac is required on the macroH2A1/H2B heterodimer or on the nucleosome-bound H2B as a genomic beacon. We generated single-chain constructs with just the HFD of macroH2A1 as we previously showed the macroH2A1 HFD was sufficient for accurate deposition (Fig. 1f) and were concerned the full length macroH2A1 protein would not fold correctly when fused to H2B. Wild-type H2B was also fused to canonical H2A as a negative control. We digested the chromatin fractions with MNase as shown previously for macroH2A1 ectopic mutants. A Flag immunoblot indicated the overwhelming majority of the ectopically expressed H2B-H2A-type single-chain proteins were predominantly chromatin incorporated (Fig. 6b).

Next, we determined the single-chain specification of deposition using ChIP-qPCR for Flag as well as endogenous macroH2A1 as a positive control. There is a strong and highly significant correlation between deposition of endogenous macroH2A1 in the H2B-H2A-Flag single-chain expressing cells with the deposition of wild-type H2B-macroH2A1^1–122^-Flag (Fig. 6c). Having established the wild-type H2B-macroH2A1^1–122^-Flag protein behaves like endogenous macroH2A1, we next determined the role of the fused H2B on macroH2A1 deposition. As indicated in Fig. 6c, there is a strong and significant correlation between the deposition of H2B-macroH2A1^1–122^-Flag with constructs containing either the WT, K15R or K20R mutation on the fused H2B. Furthermore, there is no correlation between deposition of wild-type H2B-macroH2A1^1–122_^Flag with H2B-H2A-Flag as expected from the uniform distribution of canonical H2A throughout the genome. Overall, this demonstrates that the H2B K20 in the targeted nucleosome, and not the H2B K20 in the macroH2A1/H2B dimer, is required for macroH2A1 deposition in H2Bac chromatin.

## Discussion

Our previous work demonstrated that macroH2A1 is found in two functionally distinct types of chromatin marked by either H2Bac or by H3K27me3 and that most macroH2A1-dependent transcriptional regulation occurs within its H2B-acetylated subtype^4,29^. Here, we uncover the different rules governing macroH2A1 localization to these two types of chromatin, with multiple regions being sufficient for localization of macroH2A1 to H3K27me3-marked chromatin and the carboxyl-terminal half of the HFD being required for specification of macroH2A1-containing H2B-acetylated chromatin. Previously, we demonstrated that macroH2A1.1 regulates the expression of its target genes through promotion of H2B acetylation at K12 and K120^4^. Here, we show that macroH2A1.1 alone is not sufficient for regulation of these events and instead requires its localization to the appropriate chromatin environment to perform these functions. Importantly, we identify H2BK20ac as a critical PTM governing the localization and function of macroH2A1. Overall, our results demonstrate the critical role that localization of macroH2A1 plays in the regulation of its transcriptional programs, such as the SASP.

Our results demonstrate that multiple features of macroH2A1 are sufficient to target macroH2A1 to its H3K27me3-containing chromatin environment. This result is entirely consistent with previous studies that identified dispersed elements of macroH2A1 were each sufficient to target it to the Xi^36,37^. Xist noncoding RNA triggers the formation of the highly specific, dense Xi heterochromatin structure composed of partially redundant layers of repression, including H3K27me3, DNA methylation, histone deacetylation and deposition of macroH2A1^55,56^. Xist is required for the initiation of X chromosome inactivation, but it is not required for the maintenance of the Xi. However, Xist is required to maintain the association of macroH2A1 with the Xi^57^. Since the role of Xist is largely limited to triggering heterochromatin formation on the X chromosome, future studies are required to determine if an analogous mechanism exists to target macroH2A1 to autosomal H3K27me3-containing heterochromatic regions.

**Fig. 7:**
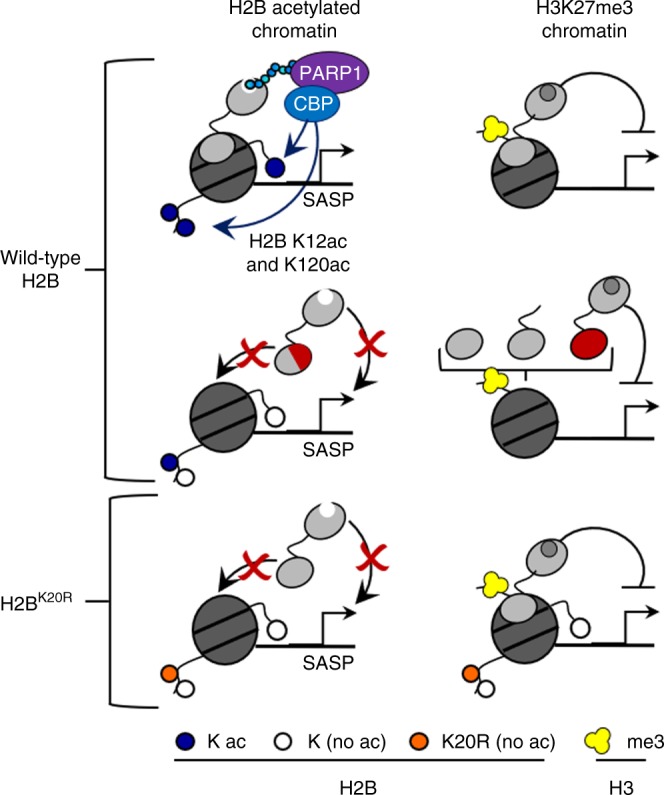
Localization to the appropriate chromatin niche is required for local macroH2A1 function. There are different rules governing macroH2A1 localization to chromatin marked by either H2Bac or by H3K27me3. Multiple regions are sufficient for the localization of macroH2A1 to H3K27me3-marked chromatin. The carboxyl-terminal half of the HFD and H2BK20ac are required for the specification of macroH2A1 to H2B-acetylated chromatin. MacroH2A1 must be localized to the appropriate chromatin environment, containing H2BK20ac as a molecular beacon, to promote downstream acetylations and regulate transcription programs such as the SASP

We show that criteria governing localization of macroH2A1 to H2Bac chromatin are distinct from those governing localization to H3K27me3-containing chromatin (Fig. 7). Specifically, the carboxyl-terminal half of the HFD of macroH2A1 is uniquely required for accurate targeting to H2Bac-marked chromatin. And, while the beacon that recruits macroH2A1 to autosomal H3K27me3-containing chromatin is unknown, our results have determined that H2BK20ac targets macroH2A1 to its H2B-acetylated environment. H2BK20ac was recently shown to be highly associated with cell-type specific promoters and more predictive of functionally active enhancers than H3K27ac^58^. H2BK20ac has also been shown to be highly correlated with H2BK120ac, especially at enhancers^58^. We previously determined that macroH2A1.1 regulates the transcription of its target genes in H2Bac chromatin through PARP1-dependent recruitment of CBP, which acetylates H2B on K12 and K120^4,29^, and now we demonstrate H2BK20ac targets its deposition to H2Bac chromatin. A key remaining goal is the identification of the upstream acetyltransferase responsible for H2BK20ac and subsequently the recruitment of macroH2A1 to H2B-acetylated chromatin.

With enhanced understanding of the key feature of macroH2A1 and the histone PTM required to specify macroH2A1-containing H2Bac chromatin, an important future goal is to identify the machinery that carries out this process. Multiple labs have performed various interaction studies to identify modulators of macroH2A1 function and deposition^3,5,59^. However, these studies have not identified a specific histone chaperone or ATP-dependent chromatin remodeling enzyme responsible for deposition of macroH2A. The exceptions come from the DNA damage field where macroH2A1 recruitment to sites of DNA damage have been reported by several groups^12,35,60–62^ where the histone chaperone APLF has been implicated and during replication stress where FACT has been implicated^35^. While the macroH2A1-deposition machinery has remained largely elusive, insights into factors that mediate the removal of macroH2A1 from chromatin have emerged. And ATRX, an ATP-dependent chromatin remodeler, has been implicated in this process^59^. In addition, our previous work demonstrated that the DNA-damage sensing kinase, ATM, promotes macroH2A1 removal specifically from H2Bac chromatin during cellular senescence resulting in its global repositioning^29^. Our recent insights into the critical features governing macroH2A1 chromatin specification could lead to new assays to identify the machinery responsible for macroH2A deposition.

The carboxyl-terminal region of H2A.Z and H2A.X have previously been identified as critical for their genomic localization^42,63^. Our results show that the carboxyl-terminal half of the HFD of macroH2A1, which harbors the docking domain, is critical for macroH2A1 localization to H2Bac chromatin. In H2A.Z, the docking domain provides specificity for its deposition and functionality^42^. In macroH2A1, the main chain of the docking domain occupies the same path as canonical H2A, despite possessing regions of divergence (Supplementary Fig. 3)^2^. Our work suggests these changes confer specificity of macroH2A1 localization to H2Bac chromatin. Binding to a chaperone can alter both the tertiary structure of the histone and the chaperone itself^64^. An additional carboxyl-terminal helix is formed in H2A.Z when bound by factors mediating its deposition, SWR1, ANP32E and YL1^44,51^. This helical region, required for H2A.Z deposition, aligns to amino acids 95–103 in macroH2A1, part of the region replaced in our mutants that are defective in localization to H2Bac chromatin. Future experiments are required to determine if the deposition defective mutant is preventing a conformational change of the macroH2A1/H2B/chaperone complex required for specific recognition and deposition into H2Bac chromatin.

Trans-histone regulatory pathways, where modification of one histone in a nucleosome (e.g., H2B K120 ubiquitination) effects a second histone modification (e.g., H3 K79 methylation), are central features of chromatin regulatory mechanisms. Histone variants also participate in trans-histone regulatory processes^65^. For example, macroH2A1.1 promotes H2BK12ac and H2BK120ac^4^. Histone variants can also be affected by upstream histone PTMs. Case-in-point, H2A.Z is targeted to sites marked by certain histone acetylations (e.g., H4K16ac, and others)^66–68^. Here, we demonstrate that H2BK20ac is an upstream PTM regulating the localization of macroH2A1 to H2B-acetylated chromatin. This result, together with the role macroH2A1 plays in promoting H2BK12ac and H2BK120ac, firmly integrates macroH2A1 as an important hub in the broader histone code.

We have operated under a model in which macroH2A1 directly regulates transcription of its target genes by cofactor recruitment and modifying local chromatin structure. There is a variety of evidence that supports this model, most notably that macroH2A1 regulated genes are typically found in or near macroH2A1-containing chromatin^4,21,29^. However, a variety of other studies failed to identify a dominant role for macroH2A1 in the regulation of gene expression^32,69,70^. Recently, an indirect, global role for macroH2A1.1 in the regulation of mitochondrial function and cellular energy metabolism has been identified^32^. By capping PAR chains, macroH2A1.1 can inhibit PARP activity reducing nuclear NAD^+^ flux and allowing a greater fraction of NAD^+^ to be available for mitochondrial respiration. This effect is global, as it does not require macroH2A1.1 to directly regulate transcription. Interestingly, recent studies of macroH2A1 knockout mice have demonstrated phenotypes consistent with altered energy metabolism^69,71^. Overall, these findings require the field to carefully re-evaluate which functions of macroH2A1 play a global role in cellular metabolism and which functions of macroH2A1 depend locally on its ability to regulate transcription. Here we demonstrate that the ability of macroH2A1 to promote H2BK12ac and H2BK120ac and regulate a key transcriptional program during senescence, the SASP, are local functions of macroH2A1 which require the carboxyl-terminal region of its HFD and H2BK20ac for its localization to target genes. These findings provide critical tools to definitively determine which of macroH2A1’s biological functions are mediated by its global effect on energy metabolism and which are a local consequence of its role in transcriptional regulation.

## Methods

### Cell culture

IMR90 (CCL-186) (ATCC) primary human fetal lung fibroblast cells were immortalized using retroviral-mediated gene transfer of the hTERT gene (Addgene plasmid 1773) (IMR90-hTERT)^14^ and maintained in MEM supplemented with 10% FBS. Human embryonic kidney 293T cells (HEK293T) (ATCC) were cultured in DMEM supplemented with 10% FBS. Cell lines in culture were routinely tested for mycoplasma contamination MycoAlert™ Mycoplasma Detection Kit (Lonza).

IMR90-hTERT cell lines expressing macroH2A1 constructs were generated using the pLVX-Puro expression system (Clontech). IMR90-hTERT cell lines expressing H2B constructs were generated using pLVX-IRES-Puro and pLVX-IRES-Neo vectors (Clontech). MacroH2A1 was cloned into and mutated within pLVX-Puro vector. H2B was cloned and mutated in pLVX-IRES-Puro and pLVX-IRES-Neo. Single chain H2B-H2A-type construct were fused directly, minus the stop codon in H2B and the methionine in the H2A-type histone. The H2B was expressed on the N-terminus and the H2A-type was on the C-terminus of the peptide. Lysine to arginine mutations were generated using standard Dpn1 mediated site-directed mutagenesis. The commercial pBABE and pBABE-H-Ras^V12^ were gifts from William Hahn (Addgene plasmid #9051). The retroviral or lentiviral infections were performed using X-tremeGENE™ 9 DNA Transfection Reagent (Roche)^4^. Three days post infection, the cells were selected for infection with 1 µg/ml puromycin and/or 0.5 mg/ml neomycin.

Paracrine senescence was induced using conditioned media from H-Ras^V12^ expressing senescent IMR90-hTERT cells (or normally growing IMR90-hTERT cells as a control) as previously described^29^. Briefly, conditioned media was collected from H-Ras^V12^-induced senescent cells or normally growing cells as a control. Starting 14 days post-infection, the cultured medium was collected from the dish every 3 days, centrifuged at 3000 rpm for 10 min, then the supernatant was filtered through a 0.2 µm pore filter (GE Healthcare). The resulting filtered medium was then mixed with an equal volume of MEM with 20% FBS and used as media for IMR90-hTERT cells expressing H2B constructs. Cells were harvested 3 days after treatment with conditioned media for RNA expression analysis.

### Immunoblots and extraction of histones

For immunoblots, histones were either acid extracted^4^ or liberated by micrococcal nuclease digestion (MNase). Briefly, cells from a 10-cm dish were lysed in 0.1% Triton X-100 lysis buffer (10 mM Tris, pH 7.9, 0.1% Triton X-100, 100 mM NaCl, 1 mM EDTA, 5% glycerol, 1 mM DTT and 1× protease-inhibitor cocktail (Roche, 11836170001)) and incubated with agitation at 4 °C for 30 min. Then, the lysate was centrifuged at 14,000 rpm for 10 min at 4 °C. The supernatant was collected as the soluble detergent lysate. For acid extraction, the insoluble pellets containing the histones were resuspended in 0.5 M HCl at 4 °C for 2 h under constant agitation. The sample was spun at 14,000 rpm for 10 min at 4 °C. The resulting supernatant containing the histones was neutralized with 2 M Tris base.

For MNase digestion, the insoluble pellet was gently resuspended in MNase digestion buffer (50 mM Tris 7.9, 25 mM KCl, 12.5% glycerol, 10 mM CaCl_2_, 4 mM MgCl_2_) and digested with MNase (NEB, M0247S) for 12 min at 37^ º^C. Digestion was stopped using MNase stop buffer (200 mM EDTA, 20 mM Tris 7.9, 0.1 mg/ml RNase). Ten microliters of the reaction was used to isolate DNA in order to confirm digestion to mononucleosomes. Briefly, the sample for DNA was cleared of protein with 0.4 mg/ml glycogen and proteinase K (2.5 U/ml, Roche) in Txn stop buffer (20 mM EDTA, 0.2 M NaCl and 1% SDS) at 37 °C for 30 min. The DNA was extracted with phenol/chloroform/isoamyl alcohol (25:24:1) and ethanol precipitated. The resultant mononucleosome DNA was confirmed by running on 1.5% agarose gel.

The acid-extracted and MNase digested fractions were used for immunoblots of histones and the detergent lysates were used for immunoblots of all other factors. The detergent lysates, acid extracts, and MNase digested material were subjected to SDS-PAGE and immunoblotting (antibody information provided in Supplementary Table 2). HRP-conjugated goat anti-mouse or anti-rabbit secondary antibody was used for detection by ECL chemiluminescence according to the manufacturer’s instructions (Thermo, Super Signal West Pico PLUS Chemiluminescent Substrate). All immunoblots have been repeated at least twice with independent biological samples. Uncropped immunoblots are provided in Supplementary Figure 7.

### Mononucleosome immunoprecipitation (MNase IP)

Three 15-cm dishes of IMR90-hTERT cells were grown to 95% confluence. The cells were collected, pelleted by centrifugation, combined by resuspending in 1 ml of 0.1% Triton X-100 lysis buffer, and incubated at 4 °C for 30 min with agitation. The nuclei were collected by centrifugation at 4000 rpm for 10 min. The nuclear pellet was then gently resuspended in MNase buffer (three pellet volumes) and digested micrococcal nuclease (5 µl NEB, M0247S) and digested at 37 °C for 10 min with gentle vortexing every 2 min. Digestion was stopped by adding MNase stop buffer (0.3 pellet volumes) and rocked at 4 °C 1.5 h to release mononucleosomes from euchromatin. The reaction was then spun at 13,000 rpm for 10 min. The supernatant contained the released mononucleosomes and the pellet had the nuclear mononucleosomes which were acid extracted as described above. Fifteen microliters of the supernatant of the MNase reaction was kept as input, and another 15 μl was used to confirm digestion to mononucleosomes (as described above). Then remaining supernatant was combined with an equal volume of HEGTw/300 buffer (20 mM Tris, pH 7.9, 1 mM EDTA, 5% glycerol, 0.1% Tween-20 and 300 mM NaCl), 40 μl protein G beads and either no antibody (negative control) or antibody against Flag. The reactions were rocked at 4 °C for 2 h. The beads were spun at 4000 rpm for 3 min and washed three times in HEGTw/300 buffer. The resulting beads were resuspended in 1X Laemli buffer, boiled, and centrifuged. The resulting supernatant containing eluted mononucleosome complexes were run on 10% or 20% SDS-polyacrylamide gels and immunoblotted for indicated proteins.

### Chromatin immunoprecipitation (ChIP)

Cells were grown to 90% confluence in 15-cm dishes, cross-linked with 1% formaldehyde in PBS at room temperature for 10 min and then quenched in 125 mM glycine for 5 min. The cells were washed with cold PBS once and were collected by centrifugation and then sonicated in lysis buffer (50 mM Tris, pH 7.9, 10 mM EDTA, 1% SDS, protease-inhibitor cocktail and 1 mM DTT) to generate chromatin fragments of ~500 bp in length. The material was clarified by 10 min centrifugation at 14,000 rpm and 4 °C, and 20 μl supernatant was used as input for quantitation. The remaining supernatant was diluted ten-fold in dilution buffer (20 mM Tris, pH 7.9, 2 mM EDTA, 150 mM NaCl, 0.5% Triton X-100, protease-inhibitor cocktail (Roche, 11836170001) and 1 mM DTT) and precleared with 20 μl protein G–agarose beads at 4 °C for 2 h. The supernatant was used in immunoprecipitations at 4 °C overnight with antibodies against macroH2A1 (8 μl), Flag (5 μl) or histone H3 (4 μl), as indicated (Supplementary Table 2) and were then incubated with 40 μl protein G-agarose beads at 4 °C for 2 h. No-antibody (NA) controls were always included. The immunoprecipitated DNA was cleared of protein by digestion with 0.4 mg/ml glycogen and proteinase K (0.45 mg/ml, Roche) in Txn stop buffer at 37 °C for 1 h. The DNA was then extracted with phenol/chloroform/isoamyl alcohol (25:24:1) and ethanol precipitated. Quantitative real-time PCR with SYBR Green (Invitrogen) was used to determine enrichment of immunoprecipitated material relative to input with gene-specific primers to the specified regions (Supplementary Table 1).

### ChIP-seq analyses

MacroH2A1 ChIP from GEO database (GSE54847) and sequencing reads for 26 additional histone marks in IMR90 cells were downloaded from the GEO website (GSE16256) and used to determine macroH2A1 enrichment. Enrichment analysis was performed as previously described^4^.

### RNA purification and RT-qPCR

mRNA levels were analyzed by reverse transcription followed by quantitative PCR (RT-qPCR). RNA was isolated with TriPure (Roche) according to the manufacturer’s protocol. The RNA was reverse transcribed with Moloney murine leukemia virus (MMLV) reverse transcriptase (Invitrogen) and a dT18 primer. cDNA, SYBR Green PCR master mix, and forward and reverse primers were used in 45 cycles of amplification (95 °C for 15 s, 60 °C for 1 minute) following 10-min incubation at 95 °C, with a LightCycler 480 (Roche). The efficiency-corrected threshold cycle (ΔCT) method was used to determine the relative levels of RNA For transcription analysis, the expression was normalized to ACTB. Melting-curve analysis was performed to ensure specificity. Primer sequences are listed in Supplementary Table 3.

## Electronic supplementary material

Supplementary Information

## Data Availability

The data that support the findings of this study are available from the corresponding author upon reasonable request.
