# Supplementary material for: MacroH2A1 chromatin specification requires its docking domain and acetylation of H2B lysine 20
Source: Nat Commun. 2018 Dec 3;9:5143. doi: 10.1038/s41467-018-07189-8 (PMC6277393; doi:10.1038/s41467-018-07189-8)
Supplement: Supplementary file 1 — Supplementary Information [file 41467_2018_7189_MOESM1_ESM.pdf]

MacroH2A1 chromatin specification requires its docking domain and acetylation of H2B lysine 20

Ruiz et al.

Supplementary Figure 1

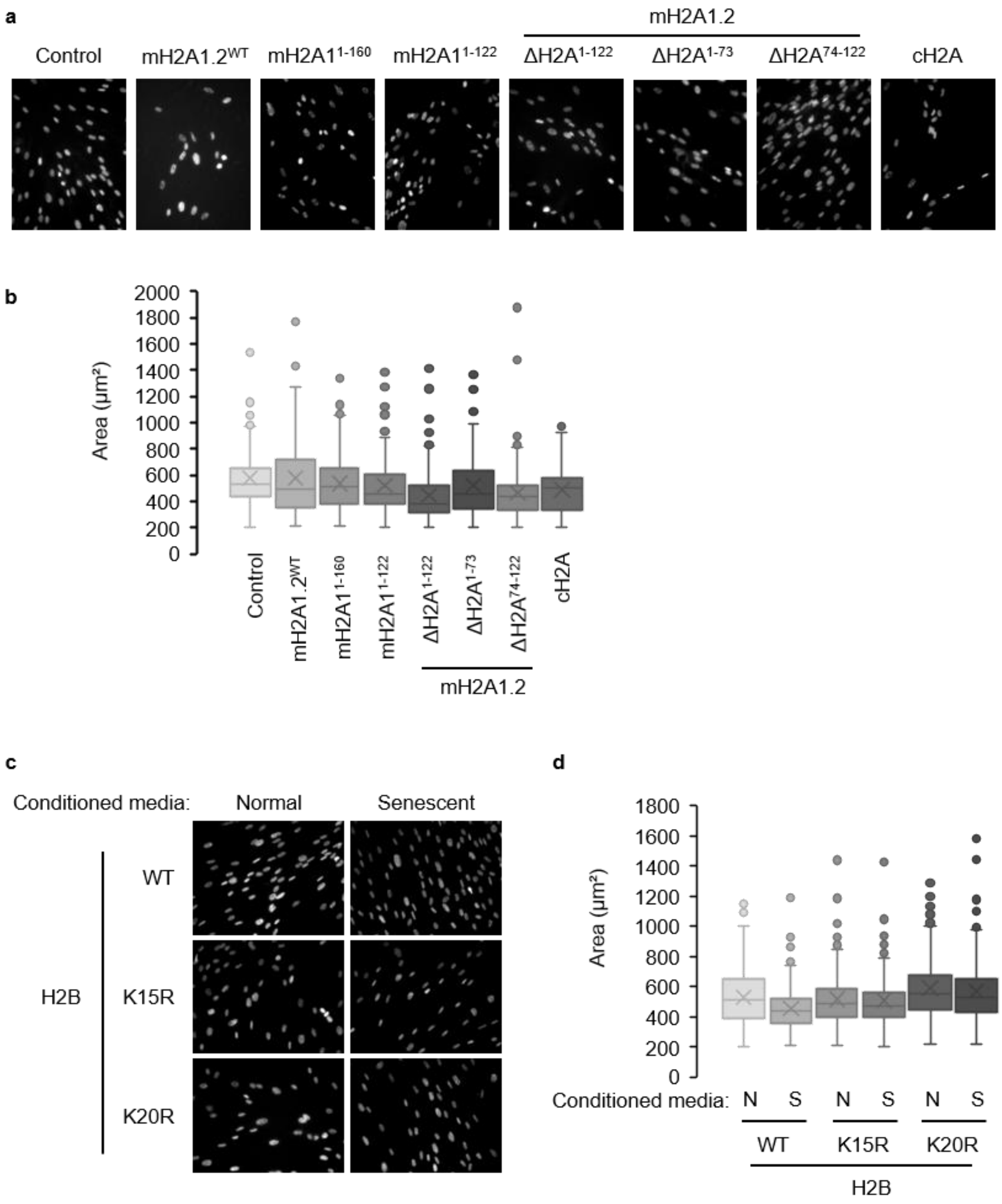

Supplementary Figure 1. Nuclear size is not affected by ectopic histone expression. (**a, b**) Representative images of indicated cells stained with DAPI and imaged using a fluorescence microscopy. (**c, d**) Nuclear size was quantified using ImageJ software. No significant differences in nuclear morphology or nuclear size were observed.

Supplementary Figure 2

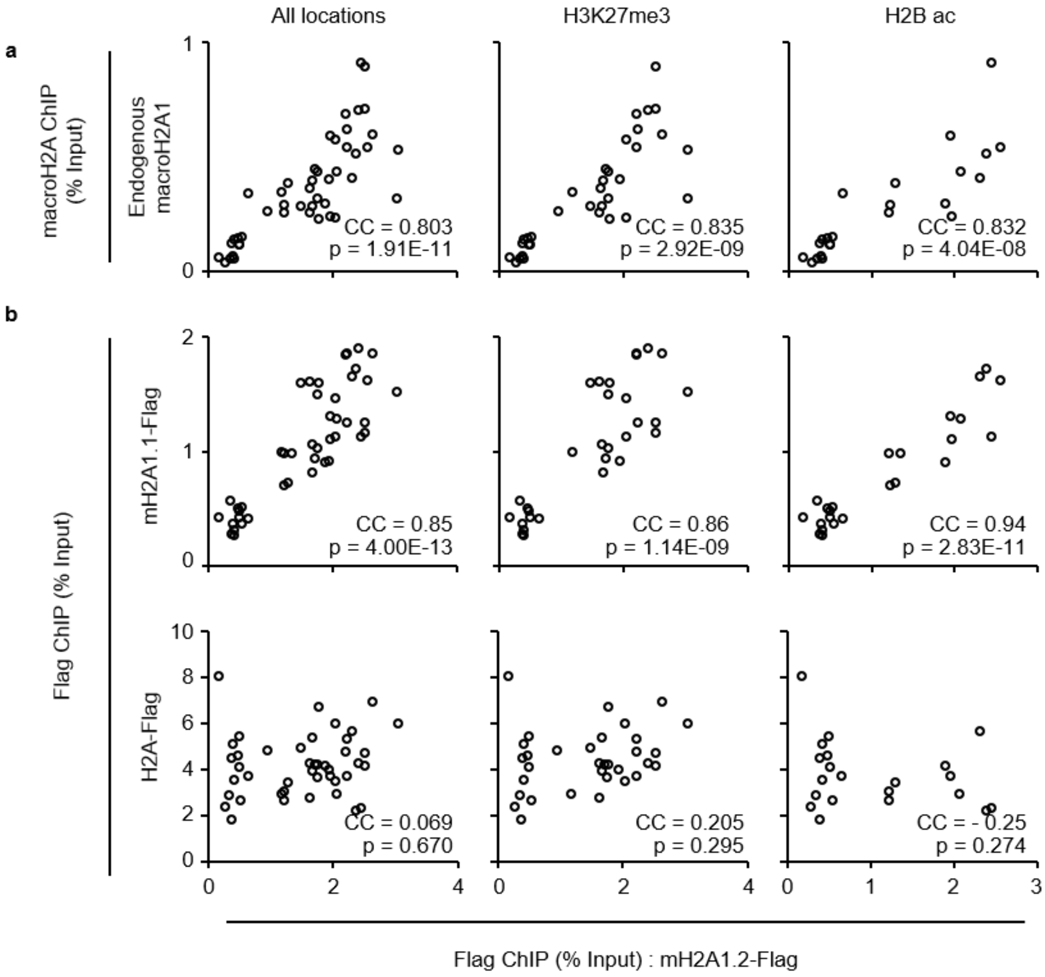

Supplementary Figure 2 (continued)

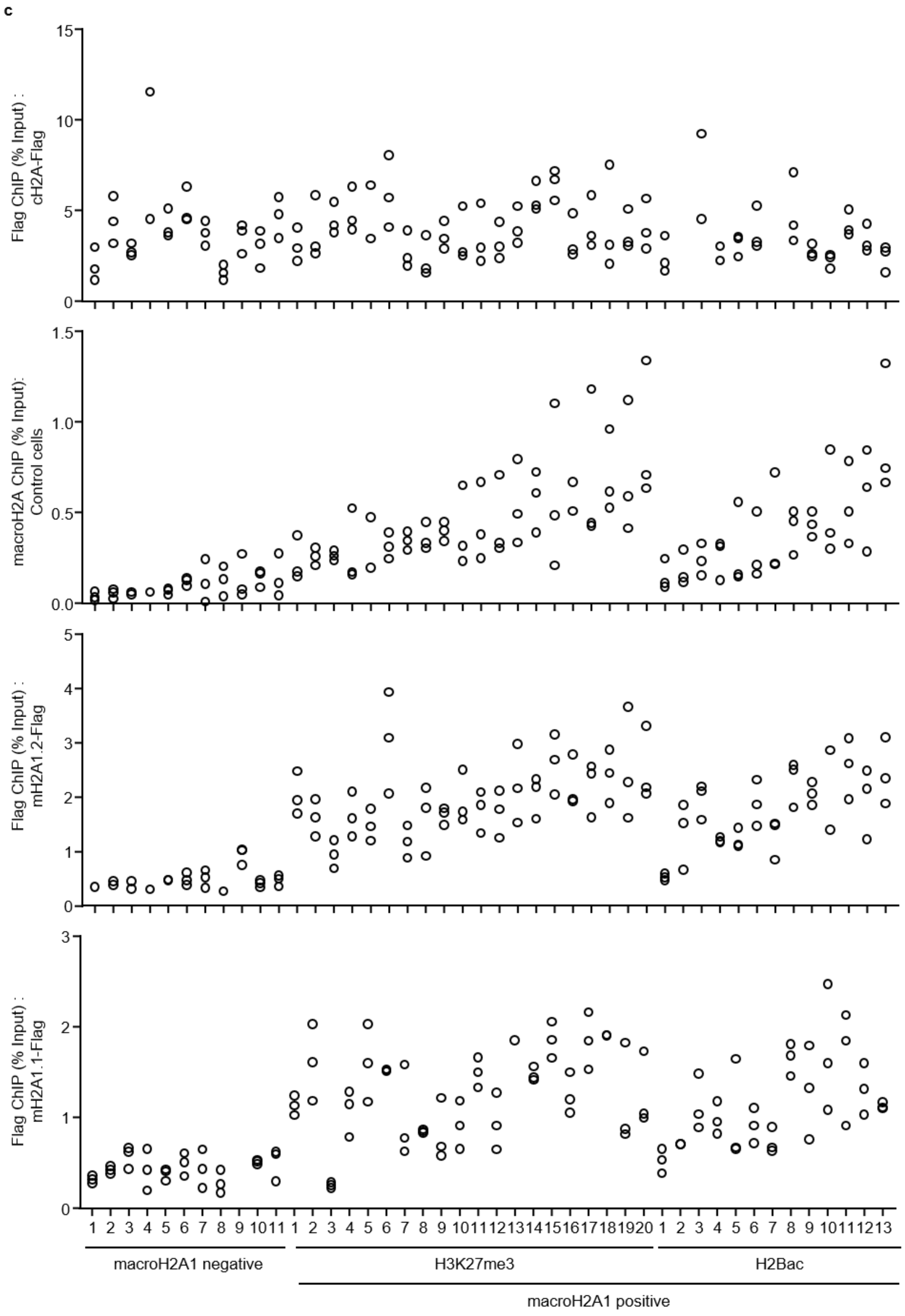

17 **Supplementary Figure 2 (continued)**

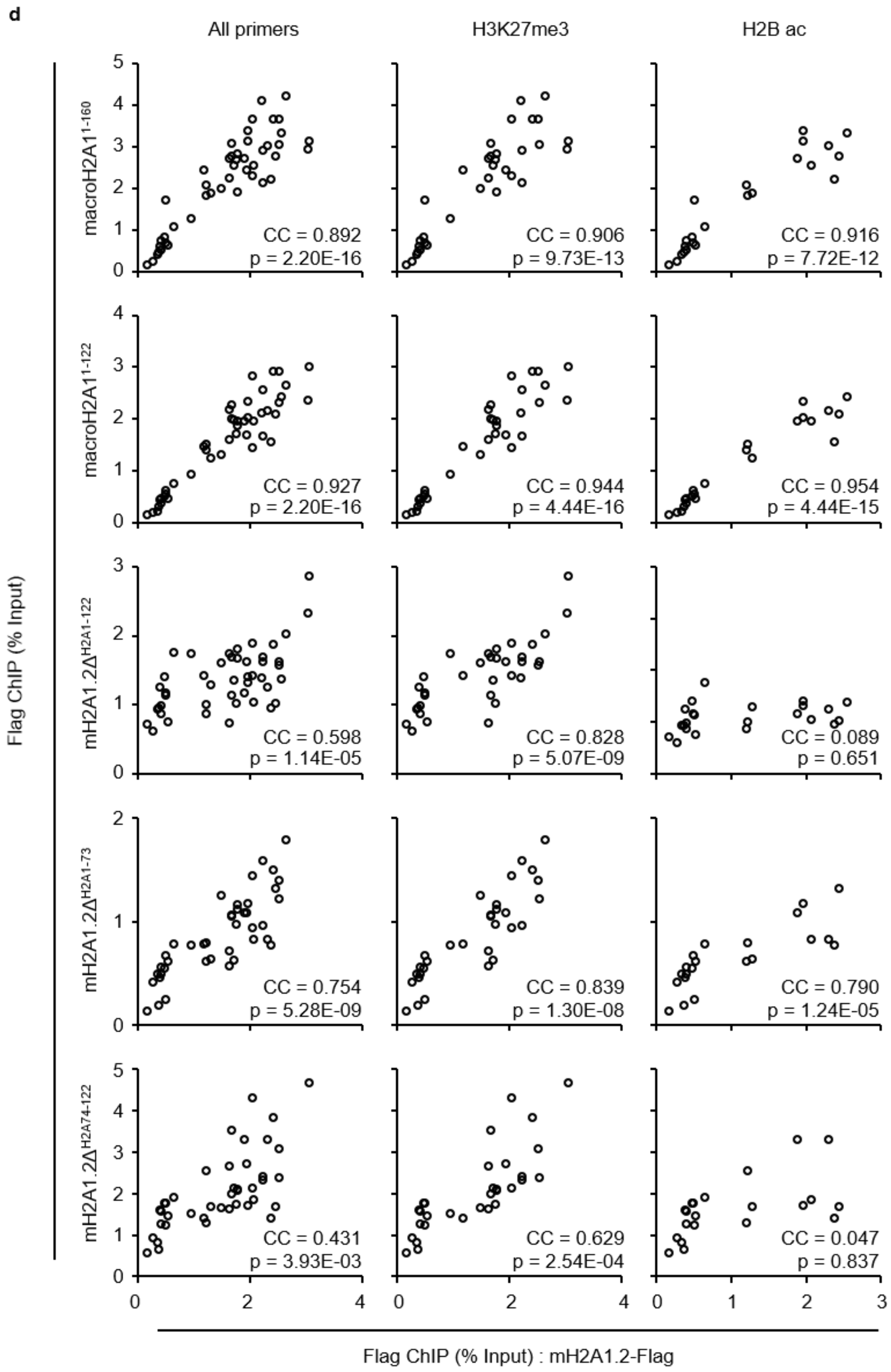

18

Supplementary Figure 2. Ectopic expression of macroH2A1 coupled with ChIP-qPCR is a robust system to monitor localization of macroH2A1. **(a)** Scatter plots of Flag ChIP-qPCR for macroH2A1.2-Flag cells vs macroH2A1 ChIP-qPCR in control cells. **(b)** Scatter plots of Flag ChIP-qPCR for macroH2A1.2-Flag cells vs macroH2A1.1-Flag cells (upper row). Scatter plots of Flag ChIP-qPCR for macroH2A1.2_Flag cells vs H2A-Flag cells. **(c)** Dispersion of data from 3 independent biological replicates for endogenous macroH2A1 ChIP in control cells and Flag ChIP for cells ectopically expressing macroH2A1.2-Flag, macroH2A1.1-Flag or canonical H2A-Flag (cH2A). **(d)** Scatter plots of Flag ChIP-qPCR for macroH2A1.2-Flag cells vs macroH2A1 mutant cells. Pearson's correlation coefficient (CC) and associated p-values calculated from Pearson's product-moment correlation are indicated for (a, b, and d). (n = 3 independent cell passages).

Supplementary Figure 3

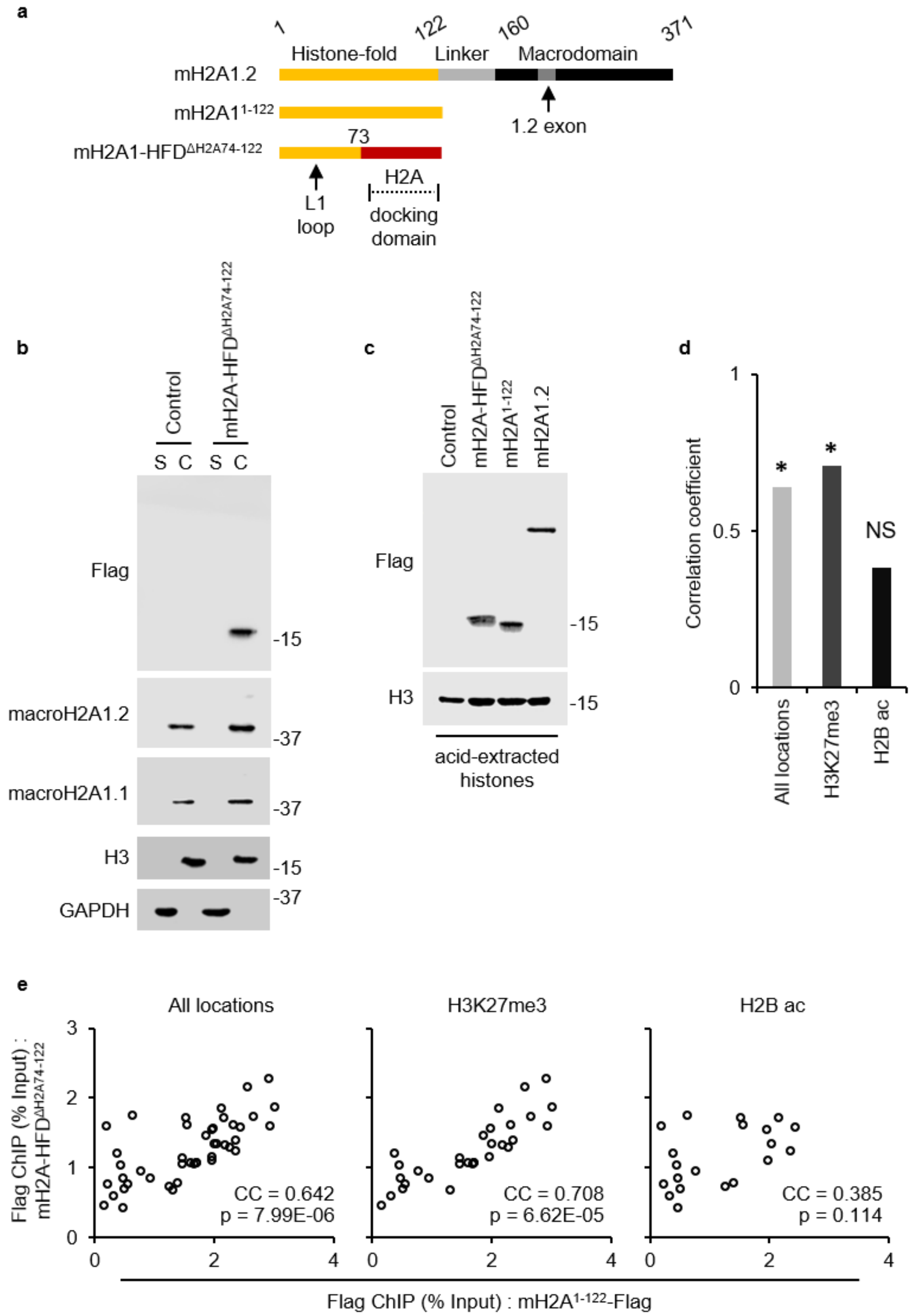

Supplementary Figure 3. The L1 loop of macroH2A1 is sufficient for accurate deposition of macroH2A1 in H3K27me3 containing chromatin. **(a)** Diagram of mutant macroH2A1HFD-Flag (mH2A1) constructs expressed in IMR90-hTERT cells. **(b)** Immunoblots of soluble (S) and chromatin (C) fractions from IMR90-hTERT cells described in (b) for indicated proteins. **(c)** Immunoblots of acid-extracted histones for indicated proteins. **(d)** Pearson's correlation coefficient calculated for Flag ChIP between macroH2A$^{1\text{-}122}$-Flag expressing cells vs. mH2A1-HFD$^{\Delta H2A74\text{-}122}$-Flag expressing cells (* $p < 1 \times 10^{-4}$) (NS = Not Significant). **(e)** Scatter plots of Flag ChIP-qPCR for macroH2A$^{1\text{-}122}$-Flag cells vs mH2A1-HFD$^{\Delta H2A74\text{-}122}$-Flag cells. Pearson's correlation coefficient (CC) and associated p-values calculated from Pearson's product-moment correlation are indicated. (n = 3 independent cell passages)

Supplementary Figure 4

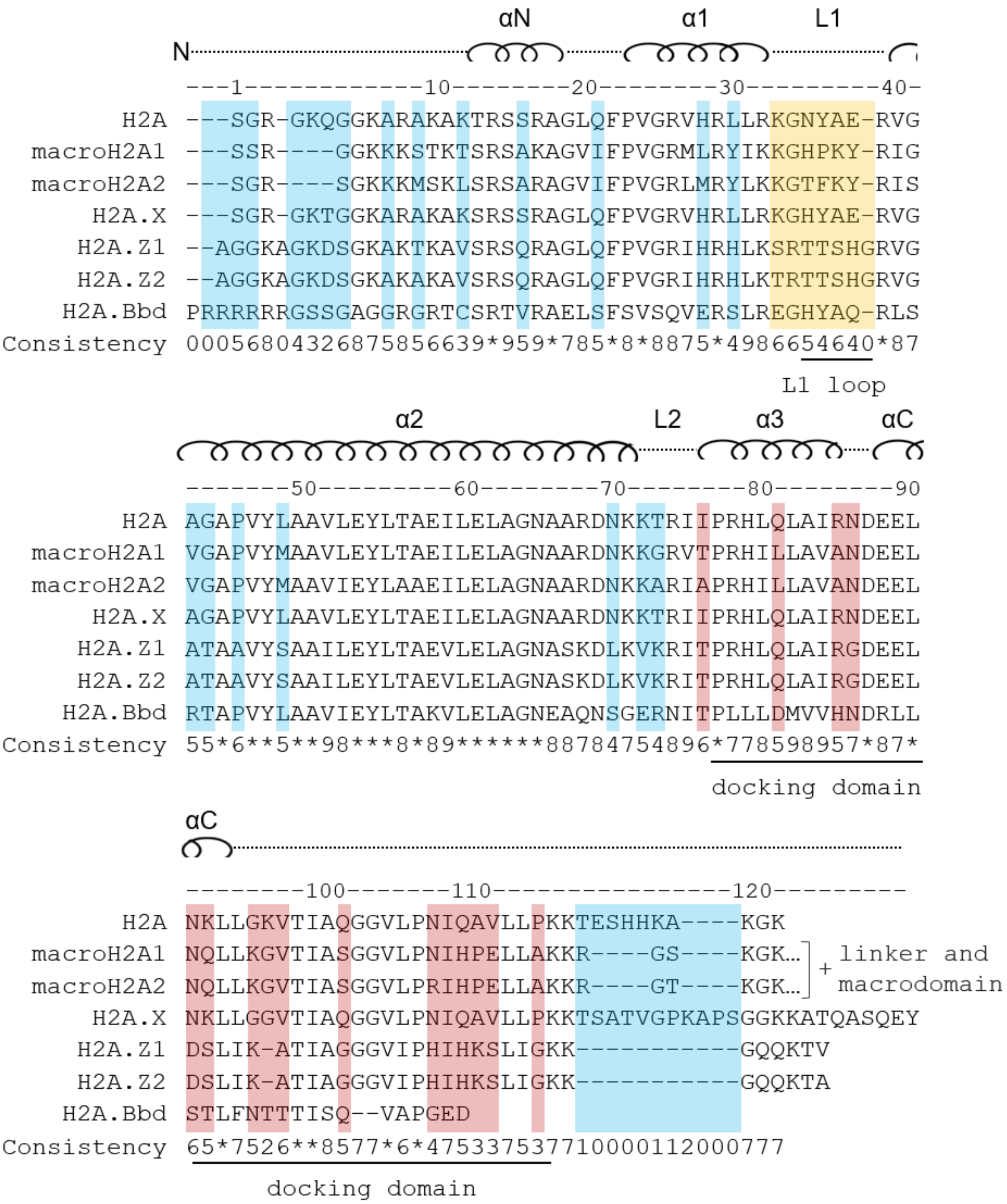

Supplementary Figure 4. Amino acid alignment of a selection of H2A-type histone variants and the histone-fold domain only of macroH2A1. Amino acid numbering is based upon macroH2A1. Regions of divergence from canonical are highlighted in yellow, red, or blue. Consistency of conservation values generated with PraLine online alignment tool.

Supplementary Figure 5

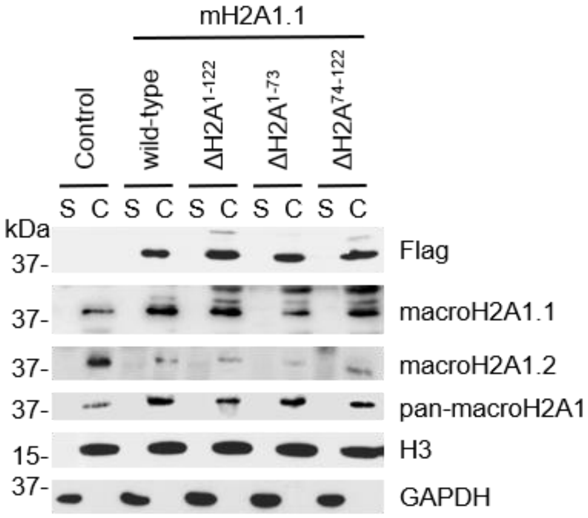

Supplementary Figure 5. Ectopically expressed macroH2A1.1 successfully incorporates into chromatin. Immunoblots of soluble and chromatin fractions from IMR90 cells described in **Fig. 2b** for indicated proteins. The chromatin-containing insoluble fraction from lysed cells was digested with micrococcal nuclease (MNase) to yield mononucleosomes.

Supplementary Figure 6

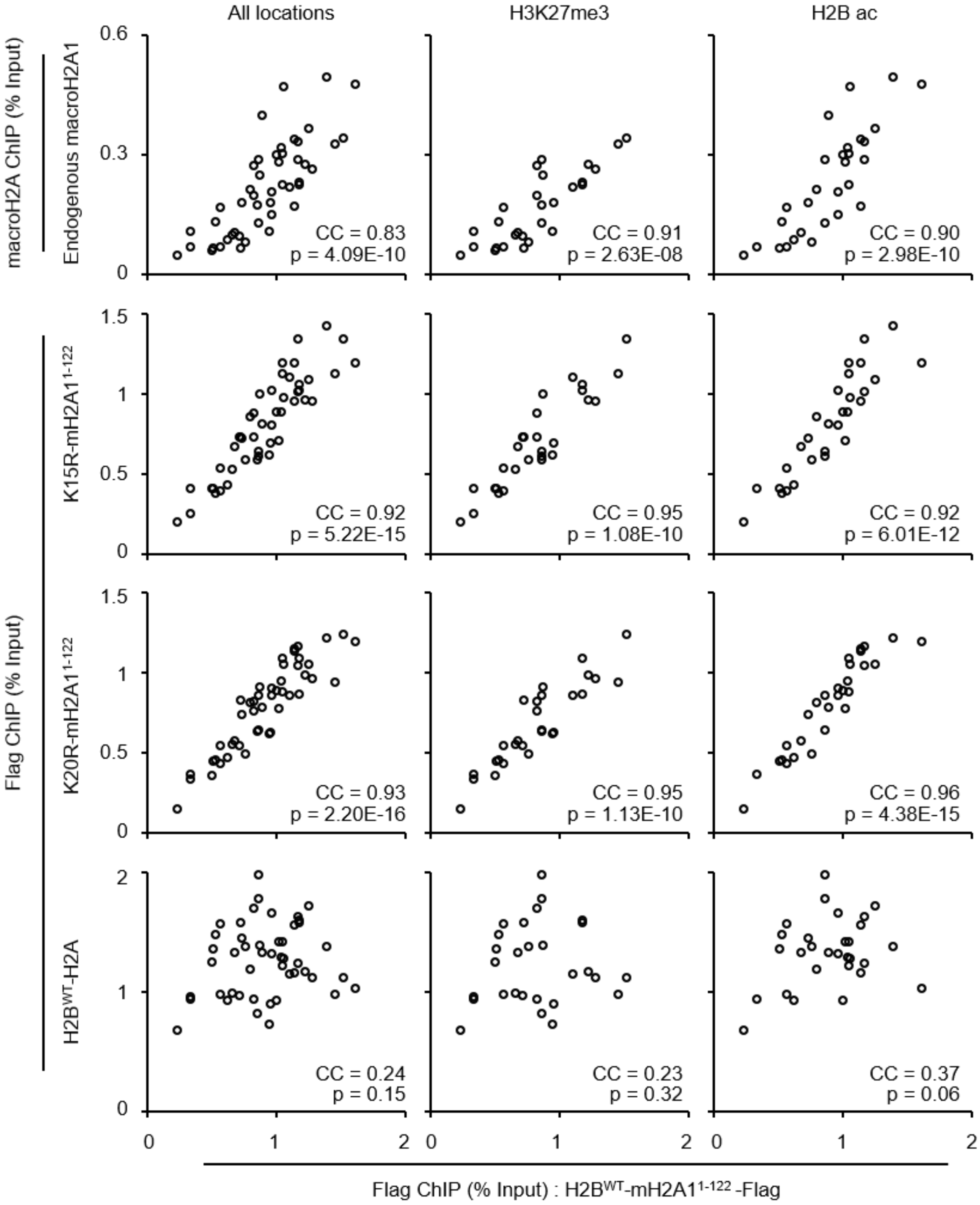

Supplementary Figure 6. H2BK20ac is not required on the obligate H2B dimer for accurate macroH2A1 localization. Scatter plots for Flag ChIP in H2B^{WT}-mH2A1$^{1-122}$-Flag expressing cells versus endogenous macroH2A1 ChIP in H2B^{WT}-H2A-Flag expressing cells and versus Flag ChIP in single chain expressing cells described in Fig. 6a. Pearson's correlation coefficient (CC) and associated p-values calculated from Pearson's product-moment correlation are indicated. (n = 3 independent cell passages).

81 **Supplementary Figure 7**

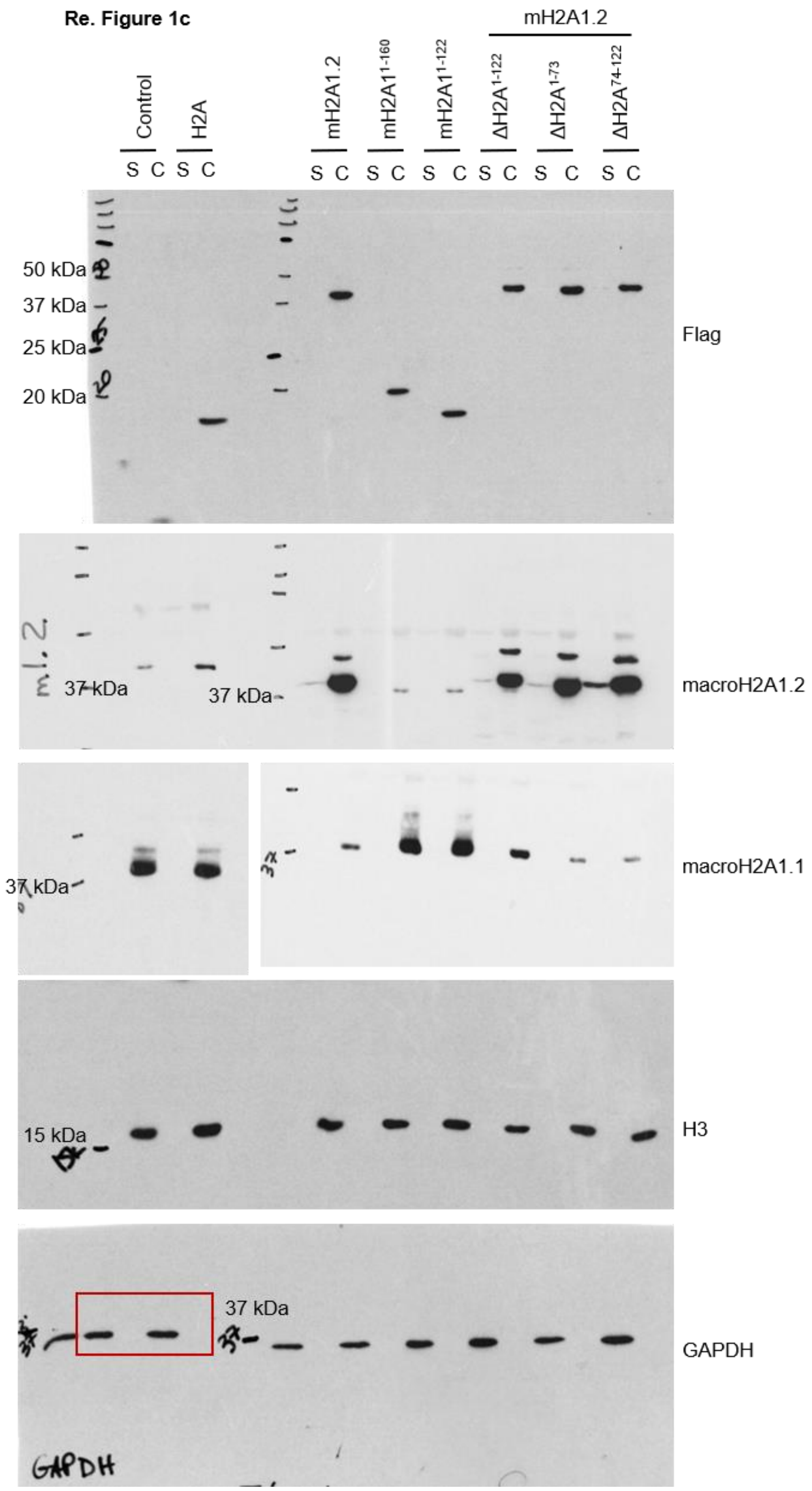

82

Supplementary Figure 7 (continued)

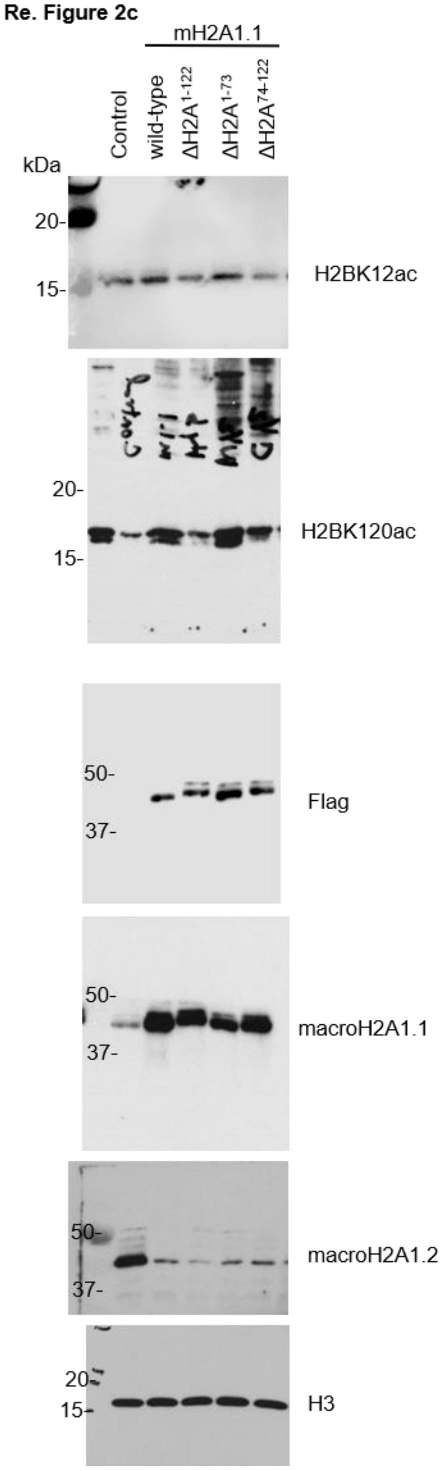

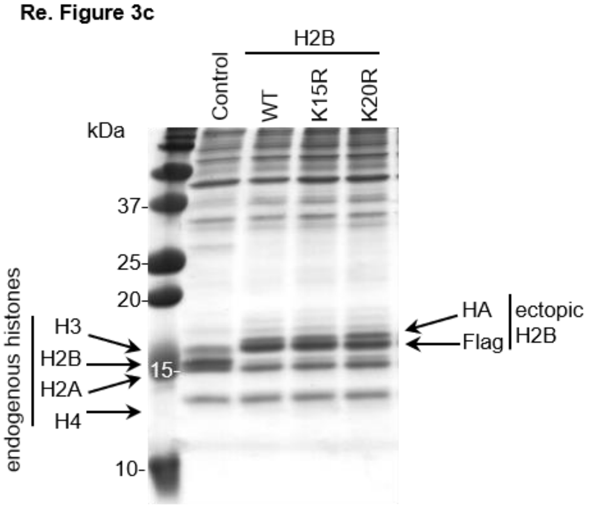

Supplementary Figure 7 (continued)

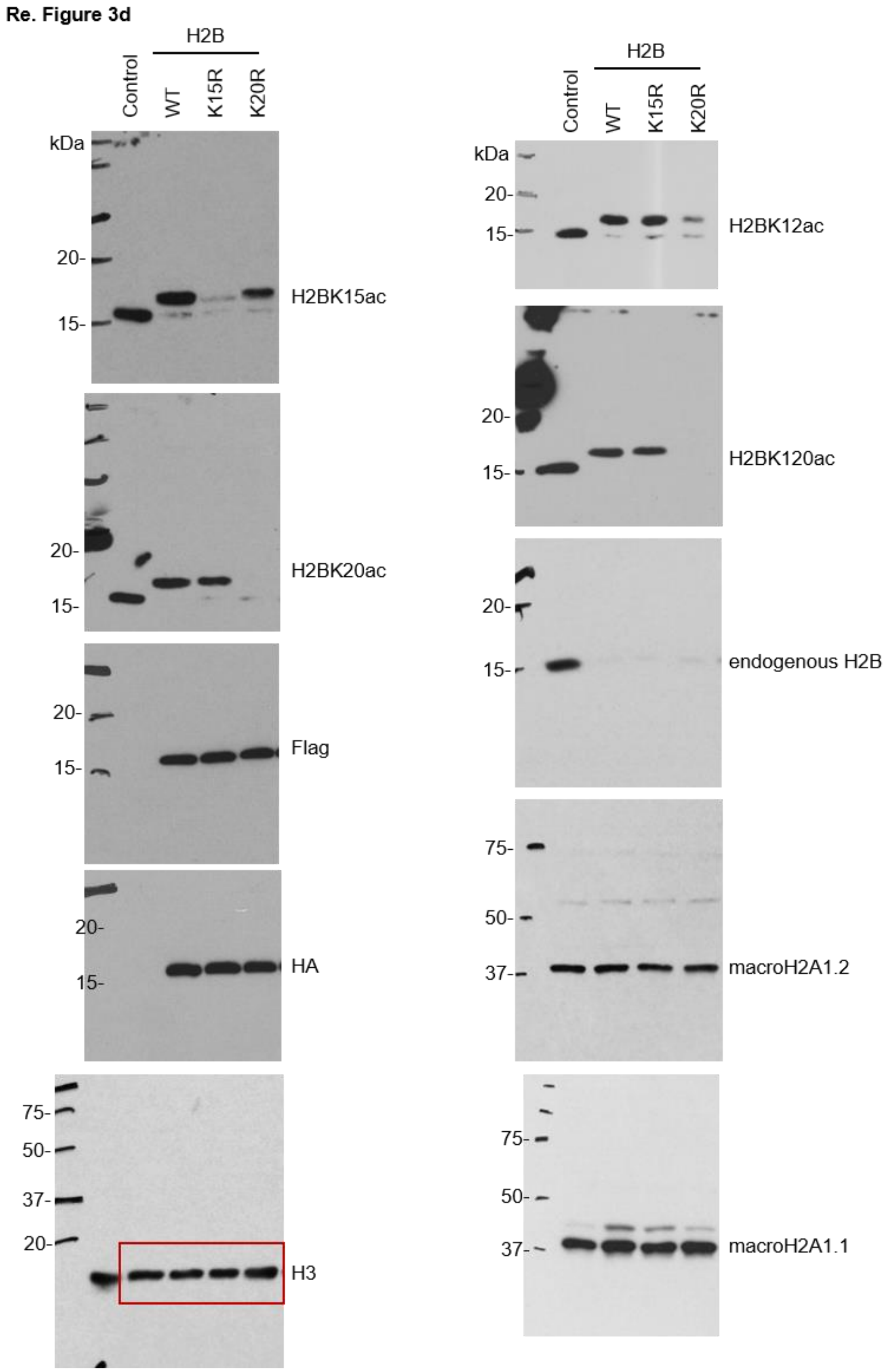

89 **Supplementary Figure 7 (continued)**

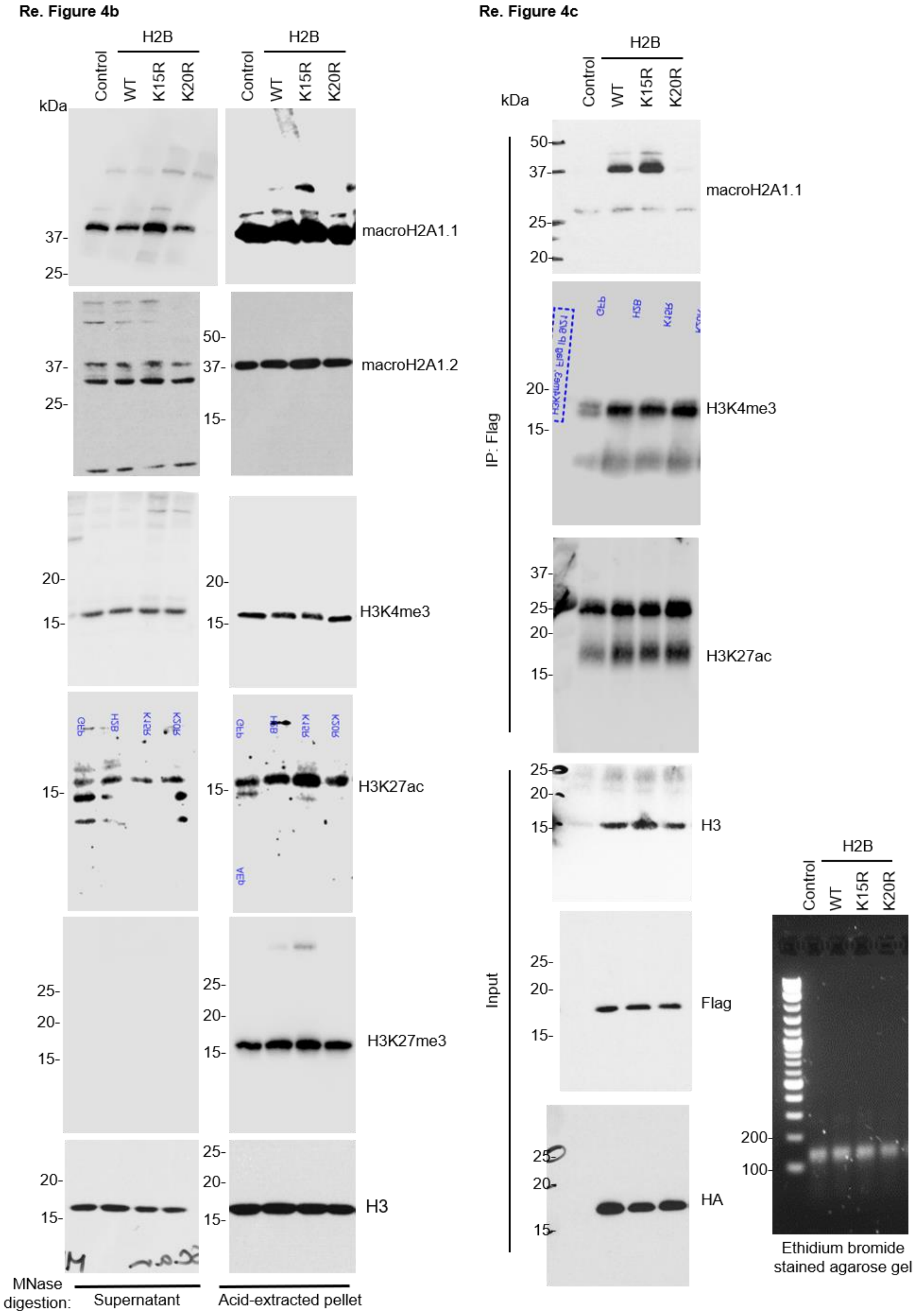

90

Supplementary Figure 7 (continued)

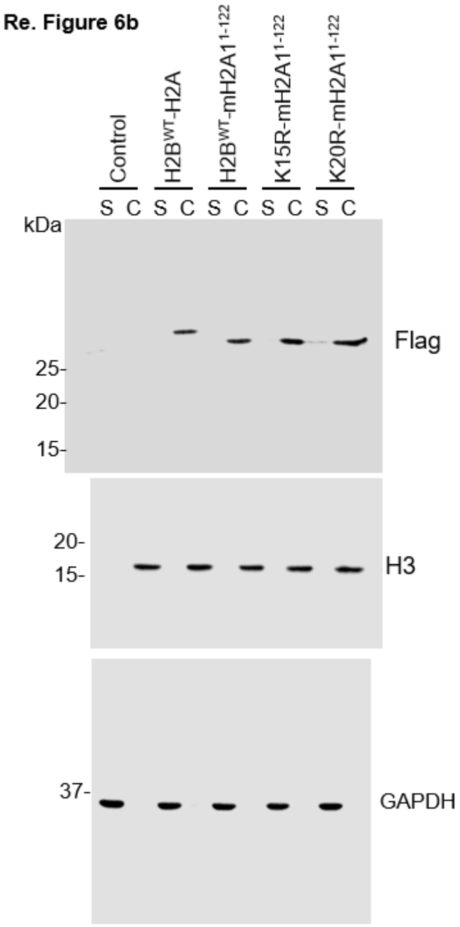

Supplementary Figure 7. Uncropped scans of immunoblots and gels for main figures are included in Supplementary Figure 7. The blots are labeled as “Re. Figure #” to indicate the related figure.

Supplementary Table 1. ChIP primers used in this study. The ChIP primers in red were used for genes indicated in Figure 2-6.

Primer set name	Location in the genome	Forward sequence	Reverse sequence	Fig 1	Fig 5	Fig 6
Unbound loci 1	chr1:52435903+52436034	AAGCCCCCTCCACTCTTCTA	AGCATGCACAGGTGTGAGAG	✓	✓	✓
Unbound loci 2	chr15:26030951+26031080	AATGGGCATCCAGTTCTCTG	CCCACCAGCTTCTTGAACTC	✓	✓	✓
Unbound loci 3	chr17:17862149+17862276	CGTACCCTCATCCCACCTTA	CCCCATCGTTTAGGGGTATT	✓	✓	✓
Unbound loci 4	chr4:41147241+41147319	TGGCATCCTGAAAAACACAA	CGGGCTTATTAGAGCAATGG	✓	✓	✓
Unbound loci 5	chr10:104502962+104503301	ATCCTGGGGGCTCTTGG	TTATTACCTCCTTCCCTC	✓	✓	✓
Unbound loci 6	chr11:94609609+94609741	TCTGGCTGTAACGTTTGACG	ATATTTGCTGTGGGGGACAG	✓	✓	✓
Unbound loci 7	chr1:54801361+54801492	GCTCATTTCTGCCTGTCCTC	AGCGACAAGAGCTGACCATT	✓	✓	✓
Unbound loci 8	chr9:14313639+14313725	GATCACCGCAACTTCACAAC	TTCACCCCTGGAAATCTAGC	✓	✓	✓
Unbound loci 9	chr7:11078494+11078603	TCCAATTGCTGTCTCCTTCA	TTAGGCCCTGATCAAACAGAA	✓		✓
Unbound loci 10	chr20:47685203+47685283	TGCTGGGTTTATGATGCACT	TTTGCATGGGTACTGCAGAG	✓		✓
Unbound loci 11	chr4:75717898+75718092	GCATCGCCAACCCTTTACTA	GCAGGGTAACCTGTGGAAAA		✓	
Unbound loci 12	chr4:78182313+78182401	ACGGACAGCTGCAGATTCTT	CTCAGCTTACCCAGCCTGAC		✓	
Unbound loci 13	chr4:152762165+152762260	CCAATAAATCCAGCCCAGAA	GGGTGCTTTTGATGGTTTGT		✓	
IL8 - 2kb	chr4:74604071+74604193	AGGCAACCGTTAGGGAAAAG	GGACACAACCTGGCTTGACT	✓	✓	✓
CXCL1 -1.6kb	chr4:74733260+74733377	TGGAAACTGAGCTTTTGGTG	TGCTACCCAACTACCCCTAATG	✓	✓	✓
CXCL6 -3kb	chr4:74699511+74699633	GCAATTTGCTGGGTGATTTT	AATTGGCCCCAAAGTGAAAT	✓	✓	✓
COL14A1+1kb	chr8:120126286+120126405	ATCTTGGTGCTGGGTTCTTG	AGAGCTGCACACAAGCTTCA	✓	✓	✓
ILRA2+1kb	chrX:114250055+114250168	ACTGGATGGGGTGAAAACAG	TTAGTGTCCGCTCCAATTCC	✓	✓	✓
KCNK2-P	chr1:215179145+215179294	ATGGGACGATGGCTTGTTAG	GAAATCCCTTTTTGCTCGTG	✓	✓	✓
NF1B-3kb	chr9:14317444+14317547	CTGGTTGCACTTTGATGACC	TTTTCCTCGCCTCACCATAC	✓	✓	✓
NFIB+2kb	chr9:14311383+14311474	TGTGGCCTCACTTTTATAACACC	ACCAGGGAAGGAATTTCAGC	✓	✓	✓
NFIB-11kb	chr9:14326083+14326166	ATGAGAGCTGTCCCTGCACTAC	TGCCTGGGTTTTCCTCTTAC	✓	✓	✓
COLEC10-2.1kb	chr8:120081391+120081493	CGGCACCAATTTATGCTTTT	GGTGCCTAGAGCACAGGAAA	✓		✓
COLEC10-3kb	chr8:120076251+120076356	GCAAAAACAGTGTTGGCTTG	AAAAATAGTGCTTCCCAGAAAGG	✓	✓	✓
COLEC10-P	chr8:120079401+120079508	GCCCTTTTTGGAATGTGTGT	TCAGAAAAGGGTCTCCCAGA	✓	✓	✓
COL14A1-33KB	chr8:121102454+121102568	GCTGTATCAGGCAGCCAGTAA	ATTTGGCCCAGCAGAGATAG	✓	✓	✓
ILI3RA-P	chrX:114252168+114252252	TTCATCCGGAAGACCGTTAG	CGGTTGCTATGGAGATGGAT	✓	✓	✓
MYC-58KB	chr8:128865201+128865332	CTTTGGCTGGCATAGTGGTT	ACCCACAGCTGATCTTGTCC	✓	✓	✓
COL14A1-1kb	chr8:121136204+121136286	TGGAATGTCGGAGGGACTAC	TTTCCAAATTTGCCTCCATC	✓	✓	
IL6-P	chr7:22767668+22767893	GATTCCTCAAAGCCATTCCA	TGGAGTCCAGAGGTGGTAGG		✓	✓
IL8+1kb	chr4:74607259+74607331	GTGCAGTTTTGCCAAGGAGT	GGGTGGAAAGGTTTGGAGTA		✓	
IL8+3kb	chr4:74609612+74609729	TGTCCTCCACAGAATGTTGG	CAGCTATGCTAAAGCGCACA		✓	
IL8-P	chr4:74606178+74606320	CATCAGTTGCAAATCGTGGA	GAAGCTTGTGTGCTCTGCTG		✓	
IL8-2kb	chr4:74604071+74604193	AGGCAACCGTTAGGGAAAAG	GGACACAACCTGGCTTGACT		✓	
MMP3-1.8kb	chr11:102715943+102716042	GGGGGAAAAACCATGTCTTG	ATTCACATCACTGCCACCAC		✓	✓
Areg+1kb	chr4:75481819+75481968	GGACAGCTGAATTTGCTTGC	AAGTAGGGTGCCTTTCGAGTC		✓	
CPA4+1kb	chr7:129934534+129934637	GCTGACTGCAGATGGTTTGA	AGCCCAGGAAGAATTCCACT		✓	
RGS4+1kb	chr1:163039747+163039884	AGGTTTGGCTCCATCATCAG	TGGGGCAGAGAGATAAGGAA		✓	
SH2CA3-1kb	chr4:42398956+42399037	TCCCAGAAAAAGTGGAAACG	AAGGTTCAGGACCGAGAACA		✓	
HSD17B2+1kb	chr16:82069648+82069795	ATCATTGTTTGGCCAGGTTC	AGCCAAGCAAACTGCAAAGT		✓	
Col14A1-P	chr8:121136993+121137067	CACAGCACCTGAGAACAGGA	GCTACTGCAAGCTCCCAAAC		✓	
ANKRD1 +1kb	chr10:92680144+92680225	CTCGTCCTGGGTCATTCTTC	ACTGCTCAAAGGTGGCTGTT		✓	
HDAC1+1kb	chr7:18536470+18536580	CATCAGCCCCAAATGAAACT	CACATCTGCCTGACAATGCT		✓	
MMP3-P	chr11:102714309+102714451	TCTATGCCTTGCTGTCTTGC	GTTTGGAAATGGTCCTGCTG		✓	
HDAC9-P	chr7:18535099+18535185	GCAAGCTTCGAGAGAGGAAA	CCAGCTGTCCAGCCTCTATC		✓	
HDAC9-1KB	chr7:18533967+18534077	TGCCCCCTCTGACTTCATAC	TCAGAAGCAACAGGGGAAAG		✓	

COLEC10-P	chr8:120079401+120079508	GCCCTTTTTGGAATGTGTGT	TCAGAAAAGGGTCTCCCAGA		✓	
LHX9+P	chr1:197881698+197881837	TGTGCCAGGTTCCTTCTGAT	TACGAAGCGCGGTTTAATTT		✓	✓
KCNK2-P	chrX:114252168+114252252	TTCATCCGGAAGACCGTTAG	CGGTTGCTATGGAGATGGAT		✓	✓
CHURC1+4kb	chr14:65376741+65376882	CCAGCAATTAGGGCCATTT	CTGCCCCTATGGGTCTCTAA	✓	✓	✓
CWC22+30kb	chr2:180902465+180902566	GCTGCAGAAGGGAAAACAAA	AGAGAGAGCTGGGGCTTGAT	✓	✓	✓
MTUS1-58kb	chr8:17571852+17571935	CATGTTAAGGAGGATGCTGTGA	CCTGCATGAAACTCTTTGGA	✓	✓	✓
MYOM2+16kb	chr8:1976627+1976699	TGATGTCTCACTCGCTGAGG	CAGGAAATCGACAGTGACCA	✓	✓	✓
PALM2+36kb	chr9:112366987+112367181	AAGCGGGCTAATGGAGAAATA	TGAGGCAGAACAATCACTGG	✓	✓	
PDCD4+16kb	chr10:112614870+112614982	TTCCCAATTCCCCCTTTTAG	GAGTTGGCCTGGAACGTAAA	✓	✓	✓
PLEKHG+3kb	chr14:65168383+65168499	GGGGGATTTTCTCCATGTTT	GAGAGGGAGGGAACGTAAGC	✓	✓	
TTC30B+22kb	chr2:178436564+178436688	AGGAGCTTGGTGGCTACAGA	TGCAAGACTGGTGCAGGATA	✓	✓	✓
PCDH9-58kb	chr13:66818356+66818519	CCCGATCAGTGTCCTGAAAG	GCTGGCAATGAAATGTCAGAT	✓	✓	✓
PHF14+100KB	chr7:10910373+10910446	GCATTGACTTTGTGTCCTTCC	GGGCATAAGGCAGTTGCTAA	✓	✓	✓
TMPRSS15+P	chr21:19775839+19775944	CCAGCACAGAGCACTACCAA	CAAAAATGGGGTCGAAAAGA	✓	✓	
SPTLC3-88KB	chr20:13077917+13078025	AGAGCAGCAGGACTTTCAGC	GGGGAACAGCATGGTAAGAA	✓	✓	
SLC2A7+P	chr1:9086648+9086738	CGAAAGCTCCCTAGTTGCAC	AGCCTCACTGGGTCTCTGAA	✓	✓	✓
ECHD3+1kb	chr10:11783025+11783215	ACATGAAAGGAATGGGAACG	CTTTCAACATTCGGGGTTGT	✓		
CYP27C1+3kb	chr2:127981025+127981137	ACAATCGCCTTGCTCTAGGA	CACGTCCAGAATGGAATGAA	✓		✓
TMEM8B+P	chr9:35854552+35854621	CAGTCTGATCCCGCTCATCT	GGAATTGGGGAATAGGCATT	✓		✓
SLC2A9-90KB	chr4:9931992+9932096	GGGAGAGCCAGAAAGTCCTC	CGTACCAATCACAGCACACC	✓		✓
GRIN2B+150BP	chr12:14133215+14133360	GGAGGCTCTGTGTGGAGAAG	CGGAATGCATTTTTCTCACC	✓		✓
ADRA1A+P	chr8:26721863+26721941	GTAGACGCGGCAGTACATGA	GGGCTACGTGCTCTTCTCAG	✓		✓
WEE2+P	chr7:141408432+141408515	GAGGGAAATTCAGGCTACCG	AAGGGAACGCTGTGAACCTA	✓		✓
MIR1244-800BP	chr12:9392983+9393075	GGACTCTGGCGATAAAGCTG	AAAAGCCGTCTTTGCATTGT	✓		✓
BC039377+P	chr21:23381588+23381682	CACCTTGCACGTCAGAAAAA	TGCAGGTCATCCTACATTGG	✓		✓

Supplementary Table 2. Antibodies used in this study.

Factor or PTM	Vendor	Cat Number	Dilution For blotting	Vol.for ChIP (µl)
macroH2A1	Millipore	07-219	1:1,000	8
macroH2A1	Millipore	ABE215	1:1,000	8
macroH2A1.1	Cell signaling	4160S	1:1,000	
macroH2A1.2	Cell signaling	4827S	1:1,000	
H2BK12ac	Aviva	OAAF08175	1:10,000	
H2BK120ac	Millipore	07-564	1:1,000	
H2BK15ac	Abcam	ab62335	1:1,000	
H2BK20ac	Millipore	07-347	1:1,000	
H3K4me3	Millipore	04-745	1:1,000	
H3K27ac	Active Motif	39135	1:1,000	
H3K27me3	Millipore	07-449	1:3,000	
H2B	Proteintech	N/A	1:10,000	
H3	Abcam	ab1791	1:3,000	4
Anti-Flag M2	Sigma	F3165	1:6,000	5
Anti-HA.11 Clone 16B12	Covance	MMS-101P	1:6,000	
GAPDH	Cell signaling	2118L	1:1,000	
HRP-conjugated goat anti-rabbit secondary antibody	Jackson Labs	111035003	1:30,000	
HRP-conjugated goat anti-mouse secondary antibody	Jackson Labs	115036003	1:30,000	
Mouse Anti Rabbit IgG, Light Chain Specific Secondary antibody	Jackson Labs	112692	1:30,000	

Supplementary Table 3. RT-PCR primers used in this study.

Gene name	Forward sequence	Reverse sequence
ACTB	AGCTACGAGCTGCCTGAC	AAGGTAGTTTCGTGGATGC
IL8	ATGACTTCCAAGCTGGCCGTG	TGTGTTGGCGCAGTGTGGTC
CXCL1	CACCCCAAGAACATCCAAAG	TAACTATGGGGGATGCAGGA
CXCL6	TGTTTACGCGTTACGCTGAG	AACTTGCTTCCCGTTCTTCA
